# Supplementary material for: Competitiveness analysis of fresh tomatoes in Indonesia: Turning comparative advantage into competitive advantage
Source: PLoS One. 2023 Nov 30;18(11):e0294980. doi: 10.1371/journal.pone.0294980 (PMC10688876; doi:10.1371/journal.pone.0294980)
Supplement: S1 Appendix — Allocation of costs into components of tradable inputs and domestic factors of tomato farming in production centres in Indonesia, 2019–2020; Appendix 2. Shadow Prices of Input and Output of Tomato Farming in Indonesia, Rainy Season, 2019–2020 (IDR/unit); Appendix 3. Shadow Prices of Tomato Farming Inputs and Outputs in Indonesia, Dry Season, 2020 (IDR/unit); Appendix 4. The Results of the Policy Analysis Matrix (PAM) Analysis of Tomato Farming in Bandung Regency, West Java, Rainy Season, 2019/2020; Appendix 5. Results of Policy Analysis Matrix (PAM) tomato farming in Bandung Regency, West Java, Dry Season, 2020; Appendix 6. Results of the Policy Analysis Matrix (PAM) for tomato farming in Banjarnegara Regency, Central Java, The rainy season, 2019/2020; Appendix 7. The Results of the Policy Analysis Matrix (PAM) for tomato farming in Banjarnegara Regency, Central Java, Dry Season, 2020; Appendix 8. Results of the Policy Analysis Matrix analysis of tomato farming in Kediri Regency, East Java, Rainy Season, 2019/2020; Appendix 9. Results of the Policy Analysis Matrix Analysis of Tomato Farming in Kediri Regency, East Java, Dry Season, 2019/2020; Appendix 10. Results of the Policy Analysis Matrix analysis of tomato farming in Tanah Karo Regency, North Sumatera, Rainy Season, 2019/2020; Appendix 11. Results of the Policy Analysis Matrix Analysis of Tomato Farming in Tanah Karo Regency, North Sumatera, Dry Season, 2020; Appendix 12. Results of the Policy Analysis Matrix analysis of Tomato Farming in Tabalong Regency, South Kalimantan, Rainy Season, 2019/2020; Appendix 13. Results of the Policy Analysis Matrix Analysis of Tomato Farming in Tabalong Regency, South Kalimantan, Dry Season, 2020; Appendix 14. Results of the Policy Analysis Matrix analysis of tomato farming in Pinrang Regency, South Sulawesi, Rainy Season, 2029/2020; Appendix 15. Results of the Policy Analysis Matrix analysis of tomato farming in Pinrang Regency, South Sulawesi, Dry Season, 2020. (DOCX) [file pone.0294980.s001.docx]

| S1 Appendix  Appendix 1. Allocation of costs into components of tradable inputs and domestic factors  of tomato farming in production centers in Indonesia, 2019 – 2020 | | | |
| --- | --- | --- | --- |
| No. | Costs | Tradable Inputs (%) | Domestic Factor (%) |
| A | Production inputs |  |  |
| 1 | Seed | 100 | 0 |
| 2 | Organic fertilizer |  |  |
|  | a. Solid organic fertilizer | 0 | 100 |
|  | b. Liquid organic fertilizer | 0 | 100 |
| 3 | An organic fertilizer |  |  |
|  | a. Urea/ZA | 100 | 0 |
|  | b. SP-36/TSP | 100 | 0 |
|  | c. KCl/KNO3 | 100 | 0 |
|  | d. NPK/PONSKA | 100 | 0 |
|  | e. Dolomite | 0 | 100 |
| 4 | Pesticide | 100 | 0 |
| 5 | Plastic mulch | 100 | 0 |
| 6 | Stake | 0 | 100 |
| 7 | Raffia | 67 | 33 |
| B | Labor |  |  |
| 1 | Pre-Harvest | 0 | 100 |
| 2 | Harvest and transportation | 24 | 76 |
| 3 | Post-harvest | 25 | 75 |
| C | Irrigation cost | 56 | 44 |
| D | Tool depreciation cost | 23 | 77 |
| E | Others cost | 0 | 100 |
| F | Land rent | 0 | 100 |
| G | Capital interest | 0 | 100 |

| Appendix 2. Shadow Prices of Input and Output of Tomato Farming in Indonesia, Rainy Season, 2019-  2020 (IDR/unit) | | | | | | | |
| --- | --- | --- | --- | --- | --- | --- | --- |
| No | Description | Bandung | Banjar Negara | Kediri | Tanah ,Karo | Tabalong | Pinrang |
| A | Production Input |  |  |  |  |  |  |
| 1 | Seeds (IDR/kg) | 148,000 | 168,000.0 | 168,000 | 88,000 | 84,000 | 88,000 |
| 2 | Organic fertilizer (IDR/kg) |  |  |  |  |  |  |
|  | 1. a. Solid organic fertilizer | 1,000 | 500 | 500 | 500 | 650 | 630 |
|  | 1. b. Liquid organic fertilizer | 98,500 | 80,000 | 85,000 | 155,000 | 80,000 | 75,000 |
| 2 | Inorganic fertilizer and lime (IDR/kg) |  |  |  |  |  |  |
|  | a. Urea/ZA | 3,795 | 3,930 | 3945 | 3,540 | 4,015 | 3,970 |
|  | b. SP-36/TSP | 3,777 | 3,912 | 3,927 | 3,522 | 3,997 | 3,952 |
|  | c. KCl/KNO3 | 4,000 | 4,135 | 4150 | 3,745 | 4,220 | 4,175 |
|  | d. NPK | 5,420 | 5,555 | 5570 | 5,165 | 5,640 | 5,595 |
|  | e. Dolomite | 500 | 2,000 | 1,400 | 600 | 2,000 | 1,500 |
| 3 | Pesticides (IDR/ha) | 5,559,000 | 12,325,000 | 10,837,500 | 17,340,750 | 13,175,000 | 8,925,000 |
| 4 | Plastic mulch (IDR/ha) | 9,360,000.00 | 6,000,000 | 5,400,000 | 5,625,000 | 5,625,000 | 5,625,000 |
| 5 | Stake (IDR/ha) | 8,750,000.00 | 10,000,000 | 5,040,000 | 4,500,000 | 8,800,000 | 8,000,000 |
| 6 | Raffia rope (Rp/ha) | 506,000 | 180,000 | 157,500 | 1,012,500 | 180,000 | 150,000 |
| B | Labor (IDR/day) |  |  |  |  |  |  |
| 1 | Pre-Harvest | 75,000 | 70,000 | 75,000 | 82,500 | 85,000 | 80,000 |
| 2 | Harvest | 75,000 | 70,000 | 75,000 | 82,500 | 85,000 | 80,000 |
| 3 | Post-harvest | 60,000 | 55,000 | 62,500 | 67,500 | 70,000 | 65,000 |
| C | Irrigation Fee (IDR/ha/season) | 2,525,000 | 1,850,000 | 1,785,000 | 2,137,500 | 2,680,000 | 2,347,500 |
| D | Tool Depreciation Cost | 505,000 | 540,000 | 464,000 | 343,750 | 182,200 | 162,200 |
| E | Land Rent (IDR/ha/season) | 12,500,000 | 7,200,000 | 8,500,000 | 8,000,000 | 5,000,000 | 6,000,000 |
| F | Other Cost | 205,000 | 250,000 | 565,000 | 675,000 | 425,000 | 425,000 |
| G | Capital Interest (IDR/ha/season) | 1758997 | 1,554,384 | 2,231,298 | 2383871 | 1,515,422 | 1,413,676 |
| H | Tomato output (IDR/kg) | 4,735 | 4,735 | 4,735 | 4,735 | 4,735 | 4,735 |

| Appendix 3. Shadow Prices of Tomato Farming Inputs and Outputs in Indonesia, Dry Season, 2020  (IDR/unit) | | | | | | | |
| --- | --- | --- | --- | --- | --- | --- | --- |
| No | Description | Bandung | Banjar Negara | Kediri | Tanah Karo | Tabalong | Pinrang |
| A | Production Input |  |  |  |  |  |  |
| 1 | Seeds (IDR/kg) | 148,000 | 148,000 | 168,000 | 88,000 | 84,000 | 88,000 |
| 2 | Organic fertilizer (IDR/kg) |  |  |  |  |  |  |
|  | a. Solid organic fertilizer | 1,000 | 500 | 500 | 500 | 650 | 630 |
|  | b. Liquid organic fertilizer | 100,000 | 80,000 | 85,000 | 155,000 | 80,000 | 75,000 |
| 2 | Inorganic fertilizer and lime (IDR/kg) |  |  |  |  |  |  |
|  | a. Urea/ZA | 3,785 | 3,930 | 3,935 | 3,980 | 4,005 | 3,960 |
|  | b. SP-36/TSP | 3,767 | 3,912 | 3,917 | 3,962 | 3,987 | 3,942 |
|  | c. KCl/KNO3 | 3,990 | 4,135 | 4,140 | 4,185 | 4,210 | 4,165 |
|  | d. NPK | 5,410 | 5,555 | 5,560 | 5,605 | 5,630 | 5,585 |
|  | e. Dolomite | 500 | 2,000 | 1,400 | 500 | 2,000 | 1,500 |
| 3 | Pesticides (IDR/ha) | 5,631,250 | 8,457,500 | 11,795,875 | 17,983,000 | 8,925,000 | 7,437,500 |
| 4 | Plastic mulch (IDR/ha) | 450,000 | 5,625,000 | 4,650,000 | 7,500,000 | 5,625,000 | 5,625,000 |
| 5 | Stake (IDR/ha) | 8,750,000 | 14,000,000 | 4,200,000 | 4,500,000 | 8,800,000 | 8,000,000 |
| 6 | Raffia rope (Rp/ha) | 506,250 | 180,000 | 157,500 | 1,046,250 | 180000 | 150,000 |
| B | Labor (IDR/day) |  |  |  |  |  |  |
| 1 | Pre-Harvest | 75,000 | 70,000 | 75,000 | 82,500 | 85,000 | 80,000 |
| 2 | Harvest | 75,000 | 70,000 | 75,000 | 82,500 | 85,000 | 80,000 |
| 3 | Post-harvest | 60,000 | 55,000 | 60,000 | 67,500 | 70,000 | 65,000 |
| C | Irrigation Fee (IDR/ha/season) | 5,050,000 | 2,560,000 | 3,560,000 | 4,267,500 | 2,680,000 | 3,675,000 |
| D | Tool Depreciation Cost | 505,000 | 540,000 | 642,000 | 343,750 | 182,200 | 162,200 |
| E | Land Rent (IDR/ha/season) | 12,500,000 | 7,200,000 | 8,500,000 | 8,000,000 | 5,000,000 | 6,000,000 |
| F | Other Cost | 333,333 | 464,000 | 560,000 | 675,000 | 435,000 | 425,000 |
| G | Capital Interest (IDR/ha/season) | 1,732,487 | 1,667,138 | 1,431,355 | 1,772,692 | 1,494,534 | 2,282,029 |
| H | Tomato output (IDR/kg) | 4,735 | 4,735 | 4,735 | 4,735 | 4,735 | 4,735 |

| Appendix 4. The Results of the Policy Analysis Matrix (PAM) Analysis of Tomato Farming in Bandung Regency, West  Java, Rainy Season, 2019/2020 | | | | |
| --- | --- | --- | --- | --- |
| Description | Revenue (IDR/ha) | Cost (IDR/ha) | | Profit (IDR/ha) |
|  |  | *Tradable input cost* | *Domestic factor cost* |  |
| Private Price | 127,223,250 | 32,574,025 | 71,967,134 | 22,682,091 |
| Social Pricing | 151,733,075 | 28,360,415 | 71,120,660 | 52,252,000 |
| Impact of divergence and Policy | -24,509,825 | 4,213,610 | 846,474 | -29,569,910 |
| 1.  *Private Profitability* (PP) : D = A – (B + C); | | |  | 22,682,091 |
| 2.  *Social Profitability* (SP) : H = E – (F + G); | | |  | 52,252,000 |
| 3.  *Private Cost Ratio* : PCR = C/(A – B); | | |  | 0.760 |
| 4.  *Domestic Resource Cost Ratio* : DRCR = G / (E – F); | | | | 0.576 |
| 5.  *Output Transfer :* OT = A – E; | | |  | -24,509,825 |
| 6.  *Nominal Protection Coefficient on Tradable Output* : NPCO = A/E; | | | | 0.838 |
| 7.  *Input Transfer*: IT = B – F; | |  |  | 4,213,610 |
| 8.  *Nominal Protection Coefficient on Tradable Input* : NPCI = B / F; | | | | 1.149 |
| 9.  *Factor Transfer* : FT = C – G; | | |  | 846,474 |
| 10. *Effective Protection Coefficient :* EPC = (A – B) / (E – F); | | | | 0.767 |
| 11. *Net Transfer* : NT = D – H; | |  |  | -29,569,910 |
| 12. *Profitability Coefficient* L PC = D / H; | | |  | 0.434 |
| 13. Subsidy Ratio to Producer : SRP = L/E. | | |  | -0.195 |

| Appendix 5. Results of Policy Analysis Matrix (PAM) tomato farming in Bandung Regency, West Java,  Dry Season, 2020 | | | | |
| --- | --- | --- | --- | --- |
| Description | Revenue (IDR/ha) | Cost (IDR/ha) | | Profit (IDR/ha) |
|  |  | *Tradable input cost* | *Domestic factor cost* |  |
| Private Price | 126,034,125 | 26,255,350 | 72,741,853 | 27,036,922 |
| Social Pricing | 145,665,625 | 26,554,034 | 71,427,733 | 47,683,858 |
| Impact of divergence and Policy | -19,631,500 | -298,684 | 1,314,120 | -20,646,936 |
| 1.  *Private Profitability* (PP) : D = A – (B + C); | | |  | 27,036,922 |
| 2.  *Social Profitability* (SP) : H = E – (F + G); | | |  | 47,683,858 |
| 3.  *Private Cost Ratio* : PCR = C/(A – B); | | |  | 0.729 |
| 4.  *Domestic Resource Cost Ratio* : DRCR = G / (E – F); | | | | 0.600 |
| 5.  *Output Transfer :* OT = A – E; | | |  | -19,631,500 |
| 6.  *Nominal Protection Coefficient on Tradable Output* : NPCO = A/E; | | | | 0.865 |
| 7.  *Input Transfer*: IT = B – F; | |  |  | -298,684 |
| 8.  *Nominal Protection Coefficient on Tradable Input* : NPCI = B / F; | | | | 0.989 |
| 9.  *Factor Transfer* : FT = C – G; | | |  | 1,314,120 |
| 10. *Effective Protection Coefficient :* EPC = (A – B) / (E – F); | | | | 0.838 |
| 11. *Net Transfer* : NT = D – H; | |  |  | -20,646,936 |
| 12. *Profitability Coefficient* L PC = D / H; | | |  | 0.567 |
| 13. Subsidy Ratio to Producer : SRP = L/E. | | |  | -0.142 |

| Appendix 6. Results of the Policy Analysis Matrix (PAM) for tomato farming in Banjarnegara Regency, Central  Java, The rainy season, 2019/2020 | | | | |
| --- | --- | --- | --- | --- |
| Description | Revenue (IDR/ha) | Cost (IDR/ha) | | Profit (IDR/ha) |
|  |  | *Tradable input cost* | *Domestic factor cost* |  |
| Private Price | 114,332,750 | 30,550,100 | 64,122,549 | 19,660,102 |
| Social Pricing | 137,054,575 | 25,161,056 | 62,747,984 | 49,145,536 |
| Impact of divergence and Policy | -22,721,825 | 5,389,044 | 1,374,565 | -29,485,434 |
| 1.  *Private Profitability* (PP) : D = A – (B + C); | | |  | 19,660,102 |
| 2.  *Social Profitability* (SP) : H = E – (F + G); | | |  | 49,145,536 |
| 3.  *Private Cost Ratio* : PCR = C/(A – B); | | |  | 0.765 |
| 4.  *Domestic Resource Cost Ratio* : DRCR = G / (E – F); | | | | 0.561 |
| 5.  *Output Transfer :* OT = A – E; | | |  | -22,721,825 |
| 6.  *Nominal Protection Coefficient on Tradable Output* : NPCO = A/E; | | | | 0.834 |
| 7.  *Input Transfer*: IT = B – F; | |  |  | 5,389,044 |
| 8.  *Nominal Protection Coefficient on Tradable Input* : NPCI = B / F; | | | | 1.214 |
| 9.  *Factor Transfer* : FT = C – G; | | |  | 1,374,565 |
| 10. *Effective Protection Coefficient :* EPC = (A – B) / (E – F); | | | | 0.749 |
| 11. *Net Transfer* : NT = D – H; | |  |  | -29,485,434 |
| 12. *Profitability Coefficient* L PC = D / H; | | |  | 0.400 |
| 13. Subsidy Ratio to Producer : SRP = L/E. | | |  | -0.215 |

| Appendix 7. The Results of the Policy Analysis Matrix (PAM) for tomato farming in Banjarnegara Regency, Central  Java, Dry Season, 2020 | | | | |
| --- | --- | --- | --- | --- |
| Description | Revenue (IDR/ha) | Cost (IDR/ha) | | Profit (IDR/ha) |
|  |  | *Tradable input cost* | *Domestic factor cost* |  |
| Private Price | 123,205,625 | 26,760,600 | 73,015,010 | 23,430,015 |
| Social Pricing | 145,814,325 | 22,609,186 | 71,676,738 | 51,528,400 |
| Impact of divergence and Policy | -22,608,700 | 4,151,414 | 1,338,272 | -28,098,385 |
| 1.  *Private Profitability* (PP) : D = A – (B + C); | | |  | 23,430,015 |
| 2.  *Social Profitability* (SP) : H = E – (F + G); | | |  | 51,528,400 |
| 3.  *Private Cost Ratio* : PCR = C/(A – B); | | |  | 0.757 |
| 4.  *Domestic Resource Cost Ratio* : DRCR = G / (E – F); | | | | 0.582 |
| 5.  *Output Transfer :* OT = A – E; | | |  | -22,608,700 |
| 6.  *Nominal Protection Coefficient on Tradable Output* : NPCO = A/E; | | | | 0.845 |
| 7.  *Input Transfer* : IT = B – F; | |  |  | 4,151,414 |
| 8.  *Nominal Protection Coefficient on Tradable Input* : NPCI = B / F; | | | | 1.184 |
| 9.  *Factor Transfer* : FT = C – G; | | |  | 1,338,272 |
| 10. *Effective Protection Coefficient :* EPC = (A – B) / (E – F); | | | | 0.783 |
| 11. *Net Transfer* : NT = D – H; | |  |  | -28,098,385 |
| 12. *Profitability Coefficient* L PC = D / H; | | |  | 0.455 |
| 13. Subsidy Ratio to Producer : SRP = L/E. | | |  | -0.193 |

| Appendix 8. Results of the Policy Analysis Matrix analysis of tomato farming in Kediri Regency, East Java, Rainy  Season, 2019/2020 | | | | |
| --- | --- | --- | --- | --- |
| Description | Revenue (IDR/ha) | Cost (IDR/ha) | | Profit (IDR/ha) |
|  |  | *Tradable input cost* | *Domestic factor cost* |  |
| Private Price | 100,539,250 | 29,114,100 | 56,220,434 | 15,204,716 |
| Social Pricing | 130,425,575 | 24,458,535 | 55,800,035 | 50,167,005 |
| Impact of divergence and Policy | -29,886,325 | 4,655,565 | 420,398 | -34,962,289 |
| 1.  *Private Profitability* (PP) : D = A – (B + C); | | |  | 15,204,716 |
| 2.  *Social Profitability* (SP) : H = E – (F + G); | | |  | 50,167,005 |
| 3.  *Private Cost Ratio* : PCR = C/(A – B); | | |  | 0.787 |
| 4.  *Domestic Resource Cost Ratio* : DRCR = G / (E – F); | | | | 0.527 |
| 5.  *Output Transfer :* OT = A – E; | | |  | -29,886,325 |
| 6.  *Nominal Protection Coefficient on Tradable Output* : NPCO = A/E; | | | | 0.771 |
| 7.  *Input Transfer* : IT = B – F; | |  |  | 4,655,565 |
| 8.  *Nominal Protection Coefficient on Tradable Input* : NPCI = B / F; | | | | 1.190 |
| 9.  *Factor Transfer* : FT = C – G; | | |  | 420,398 |
| 10. *Effective Protection Coefficient :* EPC = (A – B) / (E – F); | | | | 0.674 |
| 11. *Net Transfer* : NT = D – H; | |  |  | -34,962,289 |
| 12. *Profitability Coefficient* L PC = D / H; | | |  | 0.303 |
| 13. Subsidy Ratio to Producer : SRP = L/E. | | |  | -0.268 |

| Appendix 9. Results of the Policy Analysis Matrix Analysis of Tomato Farming in Kediri Regency, East Java,  Dry Season, 2019/2020 | | | | |
| --- | --- | --- | --- | --- |
| Description | Revenue (IDR/ha) | Cost (IDR/ha) | | Profit (IDR/ha) |
|  |  | *Tradable input cost* | *Domestic factor cost* |  |
| Private Price | 103,997,250 | 30,641,208 | 55,395,660 | 17,960,382 |
| Social Pricing | 138,711,825 | 25,969,080 | 54,982,010 | 57,760,735 |
| Impact of divergence and Policy | -34,714,575 | 4,672,128 | 413,649 | -39,800,352 |
| 1.  *Private Profitability* (PP) : D = A – (B + C); | | |  | 17,960,382 |
| 2.  *Social Profitability* (SP) : H = E – (F + G); | | |  | 57,760,735 |
| 3.  *Private Cost Ratio* : PCR = C/(A – B); | | |  | 0.755 |
| 4.  *Domestic Resource Cost Ratio* : DRCR = G / (E – F); | | | | 0.488 |
| 5.  *Output Transfer :* OT = A – E; | | |  | -34,714,575 |
| 6.  *Nominal Protection Coefficient on Tradable Output* : NPCO = A/E; | | | | 0.750 |
| 7.  *Input Transfer* : IT = B – F; | |  |  | 4,672,128 |
| 8.  *Nominal Protection Coefficient on Tradable Input* : NPCI = B / F; | | | | 1.180 |
| 9.  *Factor Transfer* : FT = C – G; | | |  | 413,649 |
| 10. *Effective Protection Coefficient :* EPC = (A – B) / (E – F); | | | | 0.651 |
| 11. *Net Transfer* : NT = D – H; | |  |  | -39,800,352 |
| 12. *Profitability Coefficient* L PC = D / H; | | |  | 0.311 |
| 13. Subsidy Ratio to Producer : SRP = L/E. | | |  | -0.287 |

| Appendix 10. Results of the Policy Analysis Matrix analysis of tomato farming in Tanah Karo Regency,  North Sumatera, Rainy Season, 2019/2020 | | | | |
| --- | --- | --- | --- | --- |
| Description | Revenue (IDR/ha) | Cost (IDR/ha) | | Profit (IDR/ha) |
|  |  | *Tradable input cost* | *Domestic factor cost* |  |
| Private Price | 117,145,150 | 37,527,788 | 62,660,250 | 16,957,113 |
| Social Pricing | 140,425,895 | 34,301,115 | 62,579,583 | 43,545,197 |
| Impact of divergence and Policy | -23,280,745 | 3,226,673 | 80,667 | -26,588,085 |
| 1.   *Private Profitability* (PP) : D = A – (B + C); | | |  | 16,957,113 |
| 2.   *Social Profitability* (SP) : H = E – (F + G); | | |  | 43,545,197 |
| 3.   *Private Cost Ratio* : PCR = C/(A – B); | | |  | 0.787 |
| 4.   *Domestic Resource Cost Ratio* : DRCR = G / (E – F); | | | | 0.590 |
| 5.   *Output Transfer :* OT = A – E; | | |  | -23,280,745 |
| 6.   *Nominal Protection Coefficient on Tradable Output* : NPCO = A/E; | | | | 0.834 |
| 7.   *Input Transfer* : IT = B – F; | |  |  | 3,226,673 |
| 8.   *Nominal Protection Coefficient on Tradable Input* : NPCI = B / F; | | | | 1.094 |
| 9.   *Factor Transfer* : FT = C – G; | | |  | 80,667 |
| 10. *Effective Protection Coefficient :* EPC = (A – B) / (E – F); | | | | 0.750 |
| 11. *Net Transfer* : NT = D – H; | |  |  | -26,588,085 |
| 12. *Profitability Coefficient* L PC = D / H; | | |  | 0.389 |
| 13. Subsidy Ratio to Producer : SRP = L/E. | | |  | - 0.189 |

| Appendix 11. Results of the Policy Analysis Matrix Analysis of Tomato Farming in Tanah Karo Regency,  North Sumatera, Dry Season, 2020 | | | | |
| --- | --- | --- | --- | --- |
| Description | Revenue (IDR/ha) | Cost (IDR/ha) | | Profit (IDR/ha) |
|  |  | *Tradable input cost* | *Domestic factor cost* |  |
| Private Price | 121,363,550 | 23,656,688 | 80,336,994 | 17,369,869 |
| Social Pricing | 149,261,405 | 38,780,434 | 61,475,167 | 49,005,804 |
| Impact of divergence and Policy | -27,897,855 | -15,123,746 | 18,861,826 | -31,635,935 |
| 1.   *Private Profitability* (PP) : D = A – (B + C); | | |  | 17,369,869 |
| 2.   *Social Profitability* (SP) : H = E – (F + G); | | |  | 49,005,804 |
| 3.   *Private Cost Ratio* : PCR = C/(A – B); | | |  | 0.822 |
| 4.   *Domestic Resource Cost Ratio* : DRCR = G / (E – F); | | | | 0.556 |
| 5.   *Output Transfer :* OT = A – E; | | |  | -27,897,855 |
| 6.   *Nominal Protection Coefficient on Tradable Output* : NPCO = A/E; | | | | 0.813 |
| 7.   *Input Transfer* : IT = B – F; | |  |  | -15,123,746 |
| 8.   *Nominal Protection Coefficient on Tradable Input* : NPCI = B / F; | | | | 0.610 |
| 9.   *Factor Transfer* : FT = C – G; | | |  | 18,861,826 |
| 10. *Effective Protection Coefficient :* EPC = (A – B) / (E – F); | | | | 0.884 |
| 11. *Net Transfer* : NT = D – H; | |  |  | -31,635,935 |
| 12. *Profitability Coefficient* L PC = D / H; | | |  | 0.354 |
| 13. Subsidy Ratio to Producer : SRP = L/E. | | |  | - 0.212 |

| Appendix 12. Results of the Policy Analysis Matrix analysis of Tomato Farming in Tabalong Regency, South  Kalimantan, Rainy Season, 2019/2020 | | | | |
| --- | --- | --- | --- | --- |
| Description | Revenue (IDR/ha) | Cost (IDR/ha) | | Profit (IDR/ha) |
|  |  | *Tradable input cost* | *Domestic factor cost* |  |
| Private Price | 106,613,500 | 30,860,806 | 60,143,512 | 15,609,183 |
| Social Pricing | 116,907,150 | 26,286,029 | 59,419,516 | 31,201,604 |
| Impact of divergence and Policy | -10,293,650 | 4,574,777 | 723,995 | -15,592,422 |
| 1.    *Private Profitability* (PP) : D = A – (B + C); | | |  | 15,609,183 |
| 2.    *Social Profitability* (SP) : H = E – (F + G); | | |  | 31,201,604 |
| 3.    *Private Cost Ratio* : PCR = C/(A – B); | | |  | 0.794 |
| 4.    *Domestic Resource Cost Ratio* : DRCR = G / (E – F); | | | | 0.656 |
| 5.    *Output Transfer :* OT = A – E; | | |  | -10,293,650 |
| 6.    *Nominal Protection Coefficient on Tradable Output* : NPCO = A/E; | | | | 0.912 |
| 7.    *Input Transfer* : IT = B – F; | |  |  | 4,574,777 |
| 8.    *Nominal Protection Coefficient on Tradable Input* : NPCI = B / F; | | | | 1.174 |
| 9.    *Factor Transfer* : FT = C – G; | | |  | 723,995 |
| 10.  *Effective Protection Coefficient :* EPC = (A – B) / (E – F); | | | | 0.836 |
| 11.  *Net Transfer* : NT = D – H; | |  |  | -15,592,422 |
| 12.  *Profitability Coefficient* L PC = D / H; | | |  | 0.500 |
| 13. Subsidy Ratio to Producer : SRP = L/E. | | |  | - 0.133 |

| Appendix 13. Results of the Policy Analysis Matrix Analysis of Tomato Farming in Tabalong Regency, South Kalimantan,  Dry Season, 2020 | | | | |
| --- | --- | --- | --- | --- |
| Description | Revenue (IDR/ha) | Cost (IDR/ha) | | Profit (IDR/ha) |
|  |  | *Tradable input cost* | *Domestic factor cost* |  |
| Private Price | 107,555,850 | 27,061,206 | 63,166,674 | 17,327,970 |
| Social Pricing | 125,747,395 | 22,083,464 | 62,440,728 | 41,223,203 |
| Impact of divergence and Policy | -18,191,545 | 4,977,742 | 725,946 | -23,895,233 |
| 1.  *Private Profitability* (PP) : D = A – (B + C); | | |  | 17,327,970 |
| 2.  *Social Profitability* (SP) : H = E – (F + G); | | |  | 41,223,203 |
| 3.  *Private Cost Ratio* : PCR = C/(A – B); | | |  | 0.785 |
| 4.  *Domestic Resource Cost Ratio* : DRCR = G / (E – F); | | | | 0.602 |
| 5.  *Output Transfer :* OT = A – E; | | |  | -18,191,545 |
| 6.  *Nominal Protection Coefficient on Tradable Output* : NPCO = A/E; | | | | 0.855 |
| 7.  *Input Transfer* : IT = B – F; | |  |  | 4,977,742 |
| 8.  *Nominal Protection Coefficient on Tradable Input* : NPCI = B / F; | | | | 1.225 |
| 9.  *Factor Transfer* : FT = C – G; | | |  | 725,946 |
| 10. *Effective Protection Coefficient :* EPC = (A – B) / (E – F); | | | | 0.776 |
| 11. *Net Transfer* : NT = D – H; | |  |  | -23,895,233 |
| 12. *Profitability Coefficient* L PC = D / H; | | |  | 0.420 |
| 13. Subsidy Ratio to Producer : SRP = L/E. | | |  | -0.190 |

| Appendix 14. Results of the Policy Analysis Matrix analysis of tomato farming in Pinrang Regency, South Sulawesi,  Rainy Season, 2029/2020 | | | | |
| --- | --- | --- | --- | --- |
| Description | Revenue (IDR/ha) | Cost (IDR/ha) | | Profit (IDR/ha) |
|  |  | *Tradable input cost* | *Domestic factor cost* |  |
| Private Price | 99,836,250 | 31,512,106 | 56,425,412 | 11,898,733 |
| Social Pricing | 126,059,905 | 24,273,470 | 55,677,770 | 46,108,664 |
| Impact of divergence and Policy | -26,223,655 | 7,238,636 | 747,641 | -34,209,932 |
| 1.    *Private Profitability* (PP) : D = A – (B + C); | | |  | 11,898,733 |
| 2.    *Social Profitability* (SP) : H = E – (F + G); | | |  | 46,108,664 |
| 3.    *Private Cost Ratio* : PCR = C/(A – B); | | |  | 0.826 |
| 4.    *Domestic Resource Cost Ratio* : DRCR = G / (E – F); | | | | 0.547 |
| 5.    *Output Transfer :* OT = A – E; | | |  | -26,223,655 |
| 6.    *Nominal Protection Coefficient on Tradable Output* : NPCO = A/E; | | | | 0.792 |
| 7.    *Input Transfer* : IT = B – F; | |  |  | 7,238,636 |
| 8.    *Nominal Protection Coefficient on Tradable Input* : NPCI = B / F; | | | | 1.298 |
| 9.    *Factor Transfer* : FT = C – G; | | |  | 747,641 |
| 10.  *Effective Protection Coefficient :* EPC = (A – B) / (E – F); | | | | 0.671 |
| 11.  *Net Transfer* : NT = D – H; | |  |  | -34,209,932 |
| 12.  *Profitability Coefficient* L PC = D / H; | | |  | 0.258 |
| 13. Subsidy Ratio to Producer : SRP = L/E. | | |  | - 0.271 |

| Appendix 15. Results of the Policy Analysis Matrix analysis of tomato farming in Pinrang Regency, South  Sulawesi, Dry Season, 2020 | | | | |
| --- | --- | --- | --- | --- |
| Description | Revenue (IDR/ha) | Cost (IDR/ha) | | Profit (IDR/ha) |
|  |  | *Tradable input cost* | *Domestic factor cost* |  |
| Private Price | 108,368,500 | 30,437,256 | 54,790,674 | 23,140,570 |
| Social Pricing | 140,582,150 | 23,458,951 | 54,977,473 | 62,145,726 |
| Impact of divergence and Policy | -32,213,650 | 6,978,305 | -186,799 | -39,005,156 |
| 1.    *Private Profitability* (PP) : D = A – (B + C); | | |  | 23,140,570 |
| 2.    *Social Profitability* (SP) : H = E – (F + G); | | |  | 62,145,726 |
| 3.    *Private Cost Ratio* : PCR = C/(A – B); | | |  | 0.703 |
| 4.    *Domestic Resource Cost Ratio* : DRCR = G / (E – F); | | | | 0.469 |
| 5.    *Output Transfer :* OT = A – E; | | |  | -32,213,650 |
| 6.    *Nominal Protection Coefficient on Tradable Output* : NPCO = A/E; | | | | 0.771 |
| 7.    *Input Transfer* : IT = B – F; | |  |  | 6,978,305 |
| 8.    *Nominal Protection Coefficient on Tradable Input* : NPCI = B / F; | | | | 1.297 |
| 9.    *Factor Transfer* : FT = C – G; | | |  | -186,799 |
| 10.  *Effective Protection Coefficient :* EPC = (A – B) / (E – F); | | | | 0.665 |
| 11. *Net Transfer* : NT = D – H; | |  |  | -39,005,156 |
| 12. *Profitability Coefficient* L PC = D / H; | | |  | 0.372 |
| 13. Subsidy Ratio to Producer : SRP = L/E. | | |  | -0.277 |
