# Supplementary material for: Competitiveness analysis of fresh tomatoes in Indonesia: Turning comparative advantage into competitive advantage
Source: PLoS One. 2023 Nov 30;18(11):e0294980. doi: 10.1371/journal.pone.0294980 (PMC10688876; doi:10.1371/journal.pone.0294980)
Supplement: Attachment — Submitted filename: POTATO-PLOSONE_2022.pdf [file pone.0294980.s002.pdf]

## [redacted] PERNYATAAN

## *CONTRIBUTORSHIP*

Yang bertanda tangan di bawah ini, kami penulis artikel berjudul:

**Analysis of competitive and comparative advantages of potato production in Indonesia** yang diterbitkan pada **PLoS ONE 17(2): e0263633) https://doi.org/10.1371/journal.pone.0263633** menyatakan bahwa sdr/i. **Saptana, Apri Laila Sayekti, Atika Dyah Perwita, Bambang Sayaka, Endro Gunawan, Syahrul Ganda Sukmaya, Nur Qomariah Hayati, Yusuf, Sumaryanto, Muhammad Prama Yufdy, Sudi Mardianto, Atika Dian Pitaloka** adalah benar penulis artikel tersebut, dengan keterangan kontribusi sebagai berikut:

| No. | Nama | Peran Kontribusi | Tanda Tangan |
|---|---|---|---|
| 1. | Saptana | Kontributor utama | |
| 2. | Apri Laila Sayekti | Kontributor utama | |
| 3. | Atika Dyah Perwita | Kontributor utama | |
| 4. | Bambang Sayaka | Kontributor utama | |
| 5. | Endro Gunawan | Kontributor utama | |
| 6. | Syahrul Ganda Sukmaya | Kontributor utama | |
| 7. | Nur Qomariah Hayati | Kontributor utama | |
| 8. | Yusuf | Kontributor utama | |
| 9. | Sumaryanto | Kontributor utama | |

| | | | |
|---|---|---|---|
| 10. | Muhammad Prama Yufdy | Kontributor utama | 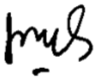 |
| 11. | Sudi Mardianto | Kontributor utama | 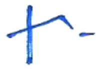 |
| 12. | Atika Dian Pitaloka | Kontributor utama | 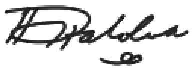 |

Demikian surat pernyataan ini dibuat dengan sebenarnya untuk dapat digunakan sesuai dengan keperluannya.

Bogor, 25 Februari 2022

RESEARCH ARTICLE

# Analysis of competitive and comparative advantages of potato production in Indonesia

Saptana[1], Apri Laila Sayekti[2], Atika Dyah Perwita[3], Bambang Sayaka[1], Endro Gunawan[1]*, Syahrul Ganda Sukmaya[4], Nur Qomariah Hayati[2], Yusuf[2], Sumaryanto[1], Muhammad Prama Yufdy[2], Sudi Mardianto[1], Atika Dian Pitaloka[5]

1 Indonesian Center for Agricultural Socio Economic and Policy Studies, Bogor, Indonesia, 2 Indonesian Center for Horticulture Research and Development, Bogor, Indonesia, 3 Faculty of Education and Teacher Training, Walisongo State Islamic University, Semarang, Indonesia, 4 Faculty of Agriculture, Universitas Jenderal Soedirman, Purwokerto, Indonesia, 5 School of Business, IPB University, Bogor, Indonesia

* endrogun007@gmail.com

Check for updates

OPEN ACCESS

**Citation:** Saptana , Sayekti AL, Perwita AD, Sayaka B, Gunawan E, Sukmaya SG, et al. (2022) Analysis of competitive and comparative advantages of potato production in Indonesia. PLoS ONE 17(2): e0263633. https://doi.org/10.1371/journal.pone.0263633

**Editor:** László Vasa, Szechenyi Istvan University: Szechenyi Istvan Egyetem, HUNGARY

**Received:** November 18, 2021

**Accepted:** January 23, 2022

**Published:** February 24, 2022

**Copyright:** © 2022 Saptana et al. This is an open access article distributed under the terms of the Creative Commons Attribution License, which permits unrestricted use, distribution, and reproduction in any medium, provided the original author and source are credited.

**Data Availability Statement:** All relevant data are within the paper and its Supporting Information files.

**Funding:** the author(s) received no specific funding for this work.

**Competing interests:** The authors have declared that no competing interests exist.

## Abstract

Fundamental issues in sustainable development of competitive potato production in Indonesia are production and distribution inefficiencies. This study aims to examine the potato production competitiveness through competitive and comparative analyses as well as evaluating the impacts of government policy on potato production. This study employs *Policy Analysis Matrix* (PAM) to analyse the cross-section data collected from six regencies in Indonesia. Potato production in Indonesia was profitable privately and socially. The highest value of competitive advantage was indicated by PCR value in the dry season in Wonosobo Regency, Central Java Province. The lowest values were found in Bandung Regency. Highest comparative advantage was revealed in Tanah Karo Regency, North Sumatra Province, during the rainy season. Highest comparative advantage was found in Bandung Regency, West Java Province, in the dry season. However, the social profit was lower than the private profit indicating the potato farmers dealt with disincentives due to imperfect market. It implies that increasing domestic potato production will be more profitable rather than import. The policy makers need to evaluate the recent policies on input and output markets as well as the supply chain of potato to cope with imperfect markets in order to increase farmers' income.

## 1. Introduction

In the era of emerging trade liberalisation, the competition among producers also increases and it creates new challenges to farmers and business actors. However, it offers new opportunities from international markets. In the demand side, trade liberalisation provides more product choices and price variations. Therefore, it may create serious problems if domestic products are unable to compete in the global market. Almost all international trade involves intermediate items, and most studies estimate that Global Value Chains (GVC) account for half or perhaps two-thirds of all global trade [1, 2].

There is research on how companies' competitiveness is defined. In this study, competitiveness was defined as a company's long-term ability to create a profit [3]. The majority of

scholars agree that competitiveness is a relative notion that may be analysed through a comparative lens [4]. Other studies show there are two indicators for measuring competitiveness in agriculture, namely farm net value and farm income [5]. The competitiveness of a product is determined by product performance in the value chain, not only in the domestic markets, but also in the global markets [6]. Domestic competitiveness is defined by actors' capacity to add value via modernising, bolstering local institutions, and enlisting industrial actors. At the global level, it is affected by the governance value chain's input-output structure, geographic breadth, and management structure connected with the leadership of businesses and industry organisations. Recent research on the value chain of agricultural goods, on the other hand, have emphasised the worldwide market, and therefore the value chain is often referred to as the GVC [7–9]. There is a vibrant growth from agricultural trade at the farm level because the role of producers in shaping their international competitiveness [10].

As an agricultural producer, Indonesia has those opportunities and challenges, but in the context of employing Regional Comprehensive Economic Partnership (RCEP), the maximum value added of agricultural products can be obtained by fully participate in regional and global value chains [11]. The Indonesia's horticultural commodities and agricultural processing industries have opportunities to participate in the value chains at the regional and global level, especially agricultural products which have high index of forward and backward linkages [12, 13]. Implementing sound value chains in horticultural commodities may stimulate economic activity in the country and ensure a more equal distribution of wealth among value chain actors [14]. Other research on GVC shows a link between GVC and competitiveness. Increased competitiveness in the global value chain can be done by 'choosing' the right partner in trade [15].

Even though there are high opportunity of horticultural products in global market, the horticultural goods in Indonesia are still in their infancy or have a low comparative advantage indicated by the high import of fruits and vegetables [16]. For example, producing potato is profitable and feasible in Indonesia, but globally it is less competitive. Potato is not a staple food in Indonesia, but the trade balance is negative particularly for food industries [17]. Indonesian potato productivity is only 17.46 tons/ha or far below the world's 50 potato-producing countries which reached 32.85–50.67 tons/ha with the highest cost per unit output in the world. Primary potato production issues are the prices of fertiliser and pesticide, as well as labour expenses, while the primary marketing issues are the low product pricing, high transportation costs, perishable items, and markets located distant from the producing area [18, 19]. The development of potato commodities in Indonesia copes with major problems, namely production, marketing, and lack of supporting policies.

Recently, however, potato export from Indonesia has been increasing along with the decreasing import [17]. In addition, potato also has backward and forward linkages. Thus, competitiveness of potato production is still arguable. The high volume of potato import is assumed for industries because the direct household consumption is only 26.72% from total consumption [17]. On the other hand, Indonesia's potato import for industrial purpose also increases [17]. A more complete and comprehensive study is needed to examine the competitive and comparative advantages of potato production in Indonesia in terms of regional distribution and growing seasons.

Typically, GVC analysis has been related to the competitiveness of products. Competitiveness analysis has been viewed from financial (private) and economic (social) perspective [18]. There are several research questions in assessing competitive and comparative advantage, as well as the impact of government policies on the value chain system: (1) Do the comparative advantages of agricultural commodities have a competitive advantage in the market? (2) What advantages do various climates offer in terms of competitiveness and comparative advantage?

(3) Does the position of competitive and comparative advantages in the existing conditions have prospects for sustainability? (4) What is the impact of government policies on the performance of farming systems? and (5) What are the incentive policy options that can change its comparative advantage into a competitive advantage in the global market?

In general, this study aims to analyse competitiveness of Indonesia's potato production. Specifically, this study aims: (i) to calculate private and social costs and profits of potato production in the producing centres; (ii) to evaluate competitive and comparative advantages of potato production in the producing centres; and (iii) to assess the impacts of government policies and strategies in order to enhance comparative advantage into competitive advantage.

## 2. Literature review

Several analyses have been used to measure competitiveness. An alternative method to measure the competitiveness status of agribusiness sector was Relative Trade Advantage/RTA [20, 21]. While the previous study employed the Dynamic Revealed Comparative Advantage (DRCA) method [22]. The Index of Revealed Comparative Advantage (RCA) has been frequently utilised to quantify competitive advantage in agricultural commodities [23–25]. However, there is an asymmetric effect of the RCA index around neutral value and must be adjusted to be symmetrical value [26, 27]. Consequently, the studies using RCA should adjust for statistical analysis, such as export, production, patent data, taxation, and international air fares [28]. Another research using Relative Export Competitiveness indexes (REC) to measure the comparative advantage of agricultural product [29]. Policy Analysis Matrix (PAM) is employed to estimate the competitiveness of agricultural commodities from the influence of policy and competitive advantages on the agricultural commodity system [30–32]. In addition, the results of the PAM analysis simultaneously produce two main indicators of competitiveness measurements. First, Private Cost Ratio (PCR) which is an indicator of competitive advantage showing the system's ability to pay domestic resource costs and remain competitive at private prices. Second, Domestic Resource Cost Ratio (DRCR) measures comparative advantage as well as the effect of government actions on the agricultural system.

Using various methods, there have been studies focusing on the competitiveness of agricultural products. The previous study concluded that there were five potential markets for agricultural products from Indonesia, namely Malaysia, Thailand, Singapore, the Philippines, and Vietnam [22]. Furthermore, it was stated that agricultural products which had opportunities to increase exports were live animals, cereals, tobacco, cocoa, and processing products including potato, especially for the Singapore and Malaysia markets. While Vietnam could be said among the biggest exporters of the world as exporting several crops in huge masses such as rice, maize and coffee [33]. Study from Van Hoang [34] reveal the ASEAN countries have the strongest competitiveness in rice, rubber, spices, vegetable fat and oils, wood, fuel wood, fish, and crustacean, and Indonesia is one of the ASEAN countries who have the strongest competitiveness in that commodity. Particularly, analysis in vegetable commodities, the previous study by [35] found that some Indonesian vegetables had a comparative advantage, but some others did not have a comparative advantage compared to the competing countries such as potato products. To increase the competitiveness of potato products, various subsystems in potato industry from providing production facilities and supporting technology, production, product quality control, post-harvest, distribution and marketing, capital and investment, as well as international trade regulations should be developed holistically and comprehensively.

In period 1999–2017, Indonesian potatoes had a fairly strong competitiveness in Singapore and Malaysia, but in 1999–2003 the RCA value in Singapore decreased compared to Malaysia [36]. This indicates a decreasing competitiveness of Indonesian potato in global market. Thus,

an effort is needed to increase export competitiveness, otherwise, Singapore and Malaysia market could be supplied from the competitors, such as China, India and Russia. The key to increase the competitiveness of an industry or product is efficiency and productivity [37, 38]. Furthermore, the sources of productivity growth are at least determined by changes in technology, technical efficiency, and economic scale of business. Another research shows that increased innovation in agriculture is supported by significant technology transfer to improve the competitiveness of agricultural companies [39]. The technology planning system is potential to be used to evaluate the risk factors and provide controlling tasks [40].

Competitive advantage at the firm level is seen as part of the foundation for building a high-level performance of a company. Competitive advantage is defined as the ability of a company to improve product quality, reduce production costs, increase volume and market share, and create profits [41]. At the firm level, competitive advantage is defined as productivity growth which can be demonstrated by lower manufacturing costs or distinctive products that entice consumers to pay a premium price [42]. Therefore, a company in a particular area can compete with similar companies elsewhere [43] and is able to explore existing opportunities, neutralise all threats that arise, and is able to reduce both production costs and distribution costs [44]. Resource factors such as institutional capabilities, financial assets, and group action, and dynamic capabilities significantly affect the competitive advantage of agricultural companies in export markets [45]. To measure comparative and competitive advantages, the PAM approach can be used to analyse potato commodities. The previous study evaluating the potato competitiveness was conducted in Banjarnegara Regency, Central Java [46]. The results indicated that potato farming was competitive both in terms of competitive advantage (PCR = 0.852) and comparative advantage (DRCR = 0.981). In addition, the results of the study from [47] showed that the incomes of potato seed farming and potato farming were profitable and feasible to the farmers. Another study evaluating the competitiveness of potato commodities in Bandung Regency, West Java, obtained a comparative advantage with a DRCR value of 0.36 and a competitive advantage with a PCR value of 0.24 [48]. This means that potato farming in Bandung Regency had very high comparative and competitive advantages with the DRCR and PCR indicator values far below one, but in fact Indonesia is a net importer of potato. However, those studies typically only evaluate partial areas of Indonesia and its potato production competitiveness is still arguable.

There are several variables associated with potato competitiveness. The previous study conducted by [49] mentioned the effects of production, inflation and exchange rates on Indonesian potato exports. The simultaneous variables of production, inflation, and exchange rates have significant effects on Indonesian potato exports in the 1993–2013 period. Partially, the potato production variable and the exchange rate have positive and significant effects, while the inflation variable has no effect on Indonesian potato exports for the said period. The findings underlined by [50] revealing that irrigated land and exchange rate had significant positive impact on agricultural product export competitiveness. In particular, it was stated that the variable dominantly influence most on Indonesian potato exports in the 1993–2013 period was potato production [49]. Therefore, it is very important to Indonesia to explore new sources of growth in potato production by expanding its harvested area and yield. One of the efforts to increase the yield of potato crop is to improve soil nutrition by reducing chemical fertiliser application and providing some bioinoculants [51]. Improved potato seed is also one of the keys to increasing productivity, competitiveness, farmers' income and potato production [41]. Studies on comparative and competitive advantages of potato production in other countries showed different results. A study in Baghdad Province showed that Iraq did not have both comparative and competitive advantages at regional and global levels. The DRCR indicator was far above 3.5, but had a competitive advantage with the PCR value of less than one, i.e.

0.41 [52]. These contrasting results were presumably due to government intervention in the form of subsidies, import tariffs, as well as obstructions in the distribution of potato commodity from abroad resulting in difficult entry to the Iraqi market. The results of the study from [53] revealed that the increase in potato production in the Wolaita zone, Southern Ethiopia, was constrained by a number of factors including disease, storage problems, falling prices for low quality tubers at harvest, and the lack of availability of quality potato tuber seeds. While a study in the Netherlands using the analysis of variable rate applications (VRA) showed that the investment would pay off in the practice conditions of potato farmers [54]. In addition, the application of this method reduced pesticides and nitrogen fertiliser application rates with an average VRA of about 25%. It indicated the increased production efficiency and more benefits to the environment. A study on competitive indicators of Egypt in the global market showed that Egypt ranked tenth with an average potato export quantity of 312.93 thousand tons equal to 2.58% of the world's average potato export during the 2010–2013 period [55]. However, in terms of the instability index during the study period, it was found that there was a very high volatility in the quantity of potato exports, namely the minimum value of 1.14%, in 2013 and the highest value of 51.84% in 2014. Moreover, in the biotech side, potatoes in the 21$^{st}$ century obtained the following main findings: (a) potato is an intensively managed crop, requiring irrigation, fertilisation, and application of pesticides to obtain high yields; (b) traditional breeding can take 15–20 years; (c) propagation through tissue culture to produce potato seeds is able to meet the aspects of quantity, quality and continuity of supply; (d) benefits of biotech potatoes include limited gene flow to conventionally grown crops, opportunities to increase productivity and nutritional quality significantly, reducing production costs, lowering negative impacts on the environment, and potentially increasing the marketability of potatoes [56–58]. In addition, to increase the comparative advantage, the role of technology is essential to deal with production issues. Research from [59] showed that communication mediated information and communication technology (ICT) had a significant effect on cooperative behaviour, disease control, return on investment and potato farmers' success rate in Ethiopia. Moreover, it is also important to policy makers to understand the farming household's behaviour related to their management and activities when formulating farming strategies to increase competitive advantages [60].

## 3. Methodology

### 3.1. Research framework

Initially, competitiveness is defined in terms of absolute advantage by Adam Smith in 1776 with "Trade Theory" which suggests the term "welfare" referring to a collection of endowments [61]. The theory proposes that two countries which have an absolute advantage could increase their welfare by trading between them. Then, Ricardo raises theory of "The Law of Comparative Advantage" mentioning even when a country lacks an absolute advantage compared to other countries in producing two types of goods, mutually advantageous trade can continue as long as there exist price differentials between the trading countries [61]. Moreover, trade among countries occurs because of differences in resources between the trading countries and achievement of economies of scale [62]. The abundant different resources could increase export and import among countries [63]. Thus, international free trade may improve welfare of countries through efficiency of domestic resources and market access to other countries [64]. According to Heckscher-Ohlin theory, exports of products are based on the abundance resources availability, for example, a country with plenty of labour and scare of capital tends to export labour-intensive products and import capital-intensive products and vice versa [65, 66]. However, since labours migrate easily among countries and high educated labour improve

competitiveness [67], the abundance of labour force in the future may not be significantly associated with the comparative advantages if they do not increase their skills and education of labours. Competitiveness is also defined as the ability of a sector, industry or firm to compete successfully in order to achieve sustainable growth within the global environment while earning at least the opportunity cost of return on resources employed [68]. Therefore, to increase export of a product, evaluating the competitiveness is necessary in order to take a proper policy to support the increasing competitiveness of a product.

To measure level of competitiveness of industry or a product in international market is determined by comparative and competitive advantages [38]. The comparative advantage can be considered natural factor based on resource abundance, while the competitive advantage is considered as acquired factor that can be developed or created. Operationally, to analyse comparative advantage, competitive advantage, and government policies' effects on a market system in Indonesia, it could employ a Policy Analysis Matrix (PAM) [30, 44, 46]. The application of this method for evaluating horticultural farming, especially potatoes, has been widely carried out by [46, 48, 52, 69–71].

## 3.2. Study areas

This paper analyses the comparative advantage, competitive advantage, and policy impact on the commodity system of potato production in Indonesia. Research was carried out in six potato producing regencies. Representing the most producing island, Java consisted of: (a) Bandung Regency, West Java Province; (b) Wonosobo Regency, Central Java Province; and (c) Pasuruan Regency, East Java Province. Meanwhile, representing the outside Java Island included: (a) Tanah Karo Regency, North Sumatra Province; (b) Solok Regency, West Sumatra Province; and (c) Kerinci Regency, Jambi Province. The research was conducted from January to December 2020.

## 3.3. Sampling methods

Research activities was conducted through a survey of potato farmer households and focused on group discussions (FGD). The number of sample farmers and FGD participants in each location was 10–15 respondents consisting of farmer leaders, farmer group leaders, administrators, traders, as well as agricultural extension workers and agricultural officers. The sampling method applied a stratified random sampling based on the wide scale of farming cultivation and the level of technology adoption. Farming analysis and comparative and competitive advantages were carried out per hectare and according to the growing season (dry season and rainy season).

## 3.4. Data analysis

**3.4.1. Allocation of tradable inputs and domestic factor components.** The production costs of potato production are separated into the allocation of tradable inputs and domestic factors. The first category cost is tradable inputs in the global market. The second cost is domestic factors not traded in the global market. Tradable goods have the following attributes: (a) commodities or products that are currently exported or imported from global markets; (b) commodities or products substituted easily with other types of products exported or imported from the global market; and (c) commodities or products protected by the government [30, 32, 72, 73].

Theoretically and empirically about PAM has been explained by [30–32]. PAM has been widely applied in several researches [38, 46, 48, 52, 73]. There are two approaches in allocating farming costs into tradable input costs and domestic factor components with the total and the

direct approaches. The direct approach assumes that the costs of tradable inputs, both imported and domestic traded globally, are categorised as tradable inputs components. Some of the reasons choosing this approach was the increasing of trade liberalisation. Therefore, additional demand for tradable inputs can be supplied from global markets. The direct approach of PAM analysis on potato farming has been widely applied [46, 48, 52, 73]. These studies categorised the output of fresh potatoes to be 100 percent tradable goods, while the inputs categorised to be 100 percent tradable are potato seeds, chemical fertiliser such as Urea, ZA, SP-36/TSP, KCl/KNO3, NPK, pesticides, and plastic mulch. Meanwhile, production inputs that are taken for granted to be 100 percent as domestic factor costs are solid organic fertiliser, liquid organic fertiliser, dolomite, raffia rope, stakes, labour, capital rent and interest.

Cost components of tradable inputs and domestic factor cost of transportation-related operations are based on discussions with representatives of business administration at various levels. Labour costs in the process of transporting goods are the domestic factor cost, while the transportation costs representing the rental value of transportation equipment are tradable in their component parts. Post-harvest handling costs are based on firsthand conversations with farmers and potato commodity merchants. The allocation of costs for materials is included in tradable inputs, while labour is allocated as domestic factors. S1 Table in S1 File shows the findings of cost components allocation into marketable inputs and domestic factors in potato growing.

**3.4.2. Social pricing.** Determination of private and social prices is needed to calculate the analysis of private and social feasibilities of an agricultural commodity farming system. Moreover, each input and output in the farming system is set as private prices and social prices. Private prices can be defined as the level of market prices received by producers in selling the output produced and/or the level of prices paid by producers in procuring the necessary production inputs. While social prices are prices formed in economic conditions where the market mechanism is perfectly competitive or the economy is in a state of general equilibrium (full employment) [72, 74]. Empirically, it is difficult for the equilibrium cost to be equal to the market prices and, thus, it can be approached using an opportunity cost such that social prices need to be adjusted to government policies and market distortions. The shadow price is determined by removing distortions caused by government policies, such as subsidies, import tariffs, value added taxes and government policy on the potato commodity system. This study, in which potato is a traded product, can be approached using border prices. As an illustration, for exported products, free on-board prices (FOB) are used and imported products use cost, insurance, and freight (CIF) prices with various adjustments made to the level where competition occurs between exported and imported goods. For domestic factors, it uses an opportunity cost or the average price in each sample area. The calculation method follows the method used by [73] by computing and adjusting in accordance with the location and potato farming studied:

1. The social price of potato seed is approached using the average price of potato seed in each research location based on argument that most farmers use their own seed or purchased from local seed breeders. Therefore, the average price of potato seeds needs to be reduced by value added tax of 10% to obtain the social price of potato seeds.
2. The social prices of solid organic and liquid organic fertilisers have increased in each research location with various types and various contents. Thus, they are approached using the actual average prices at the locality level.
3. The social price of Urea fertiliser is based on the FOB price which at the time of the research was conducted (2019) as much as US$ 0.134/kg, then converted to the dollar exchange rate

against the Indonesian rupiah, i.e. IDR 1,4141/U$ to IDR 1,895/kg. The next step is to calculate for each research location by considering export taxes, value added taxes, and transfer fees from the port to the farmer level so that the social price of Urea fertiliser is obtained.

4. The social price of SP-36 fertiliser is based on the CIF price of U$ 0.231/kg, then converted to the dollar exchange rate against the rupiah, which is IDR 1,4141/U$ to IDR 3,267/kg. The next step is to calculate for each location by taking into account import tariffs, value added tax, as well as transfer fees from the port to wholesalers and transfer fees from wholesalers to the farmer level so that the social price of SP-36 fertiliser is obtained.

5. The social price of KCl fertiliser is calculated using the CIF price of US$ 0.308/kg and the rupiah/dollar exchange rate, which is IDR 14,141/U$ to IDR 4,355/kg. The next step is to calculate for each research location by considering import tariffs, value added tax, as well as transfer fees from the port to wholesalers and transfer fees from wholesalers to the farmer level so that the social price of KCl fertiliser is obtained.

6. The social price of NPK fertiliser is according to the CIF price of U$ 0.401/kg, then converted to the dollar exchange rate against the rupiah, which is IDR 14,141/U$ to IDR 5,671/kg. The next step is to calculate in each research location by taking into account import tariffs, value added taxes, as well as transfer fees from the port to wholesalers and transfer fees from wholesalers to the farmer level so that the social price of NPK fertiliser is obtained.

7. For the social price of dolomite, because it is produced in each production areas and is not traded internationally, it is approached using the study location's real average price.

8. The social price of pesticides uses the real average price in each of the study locations, then reduced by import tariff of 10% and value added tax of 10%, such that the social price of pesticides is obtained.

9. The social price of plastic mulch is approached with the actual average price in each study location, then reduced by import tariff of 10% and value added tax of 10%, such that the social price of plastic mulch is obtained.

10. The stake's shadow price is calculated using the current average price in each study location, because it is produced by the farmers themselves and the materials are obtained locally, it is approached using the study location's real average price.

11. The shadow price of labour is approached by the actual wage value that applies in each research location of the potato production areas. The labour market mechanism works well with the daily wage system and piece rate system that can reflect the opportunity cost of labour.

12. Land rent values in each study location are used to estimate the social price of land, the argument is that the land market mechanism in the potato production centre area is running well, which is indicated by the operation of the land-leasing system and the profit-sharing system that reflects the opportunity cost of the land.

13. The social price of irrigation is approached by using the actual cost of irrigation that applies in each research location of potato production areas because the water market mechanism with a pump system works well with the water pump rental service.

14. The price of the social interest rate uses the real interest rate, which is calculated by subtracting the actual interest rate from the inflation rate that occurs because most potato farmers have access to BRI and BRI Unit loans, the actual interest rate uses the national

bank, i.e., BRI for rural loan with interest rate 3.31% per 4 months and inflation rate of 0.55% per month or 2.2% per 4 months such that the social price of capital interest is 1.31% per planting season (4 months).

15. The social cost of the rupiah to dollar exchange rate is carried out using the average actual exchange rate in 2019 because Indonesia follows the floating exchange rate regime. Thus, the social price of the rupiah exchange rate against the dollar is IDR 14,141/U$.
16. The price of social output of potato is based on the CIF price of U$ 0.800/kg, then converted to the dollar exchange rate against the rupiah of IDR. 14,141/U$ such that it becomes IDR 11,050/kg. The next step is to calculate for each location by considering the import tariff of 10%, value added tax of 10% as well as adding transfer costs from the port to wholesalers in the provincial city, then deducting the transportation costs from wholesalers in the provincial city to the farmer level such that it is obtained the social price of potato output.

The complete and in detailed calculations of the social prices of inputs and outputs in the potato farming system are presented in S1 File.

**3.4.3. PAM matrix construction.** The PAM computation stages consist of five steps, i.e., (1) determining the complete physical input and output of the analysed commodity farming system; (2) the cost of a product is broken down into components that may be traded in domestic elements; (3) calculate the amount of revenue; (4) estimating the social price of inputs and outputs); and (5) All the calculating and analysing various indicators resulting from the PAM analysis presented in the analysis tables in this paper (Supporting Information).

The next stage of compiling the PAM matrix is carried out after all data at the farmers' level and supply chain actors for potato commodities have been obtained. The PAM matrix is prepared by using the physical input-output structure at the farmers' level, budgeting costs and private and social revenues, as well as obtaining transportation cost data from the trading system. Based on this calculation, it can be obtained the private as well as the public benefits. The impact of government policies are applied in both inputs, outputs as well as inputs and outputs as a whole such that the magnitude are able to be seen.

Several results of PAM analysis provide information on profitability both privately and socially, comparative and competitive advantages, as well as the impact of government policies on potato commodity farming systems in terms of inputs, outputs as well as inputs and outputs as a whole. PAM matrix in each location is presented in Table 1.

## 4. Results and discussion

### 4.1. Private and social costs and profit

The most important thing to the farmers as the producers is that the business provides profit financially (privately). Based on the financial (private) profitability analysis, it was found that potato farming in dry highland production areas in Indonesia provided moderate to high profits. Meanwhile, the most important thing to the government and the public is that economic activities carried out by economic actors provide social benefits. The results of the analysis of social benefits showed that potato farming in Indonesia provides a higher level of profit compared to private profits.

The highest private financial profit (private) potato farming was found in Kerinci Regency, Jambi Province, in the dry season with a profit rate of IDR 41,532,849/ha/season, but not in the rainy season. Meanwhile, the lowest profit was found in Tanah Karo Regency, North Sumatera Regency, which was IDR 18,924,987/ha/season in the dry season but did not happen in the rainy season.

**Table 1. Policy Analysis Matrix (PAM).**

| Variables | Revenue | Cost | | Profit |
|---|---|---|---|---|
| | | *Tradable input* | *Domestic factor* | |
| Private price | A | B | C | D |
| Social Price | E | F | G | H |
| Policy and divergence impacts | I | J | K | L |

Source: [30]

I = A–E; J = B–F; K = C–G; L = D–H

1. *Private Profitability* (PP): D = A–(B + C)
2. *Social Profitability* (SP): H = E–(F + G)
3. *Private Cost Ratio*: PCR = C/(A–B)
4. *Domestic Resource Cost Ratio*: DRCR = G / (E–F)
5. *Output Transfer*: OT = A–E
6. *Nominal Protection Coefficient on Tradable Output*: NPCO = A/E
7. *Transfer Input*: IT = B–F
8. *Nominal Protection Coefficient on Tradable Input*: NPCI = B / F
9. *Transfer factor*: FT = C–G
10. *Effective Protection Coefficient*: EPC = (A–B) / (E–F)
11. *Net Transfer*: NT = D–H
12. *Profitability Coefficient* L PC = D / H
13. *Subsidy Ratio to Producer*: SRP = L/E.

https://doi.org/10.1371/journal.pone.0263633.t001

Results showed the highest economic (social) benefits of potato farming were found in Wonosobo Regency, Central Java Province, with economic (social) benefits in the rainy season reaching IDR 126,646.043/ha/season and profits in the dry season of IDR 92,122,351/ ha/season. Meanwhile, the lowest economic (social) benefit was found in Tanah Karo Regency, North Sumatra Province, in the rainy season, i.e., only IDR 65,759,612/ha/season and in the dry season, i.e., only IDR 566,695/ha/season. In Table 2, complete and detailed description of the financial (private) and economic (social) profit levels of potato farming in each research location are presented. The results of the analysis of the PAM matrix indicators are presented in Supporting Information.

## 4.2. Competitive and comparative advantages

Results showed that the competitive advantage of potato farming in production areas in the dry highlands in Indonesia had competitiveness as indicated by the PCR coefficient value <1. The competitive advantage possessed at a moderate level is reflected in the PCR value between 0.5 <1. Meanwhile, the results of the comparative advantage analysis reflect that potato farming has a moderate to high level of competitiveness. The high comparative advantage was indicated by the DRCR coefficient of 0.323–0.499. The PCR coefficient value <1 indicated that it costed one dollar in private market prices to create one additional worth of potato production, less than one unit of domestic resource costs was required. The value of DRCR<1 shows that to produce one unit of potato output at social prices, the domestic resource costs are less than one unit.

During the dry season, the region of Wonosobo Regency in Central Java Province had the lowest PCR coefficient value for potato production. It indicated a significant competitive advantage, but it was not achieved in the rainy season. The lowest DRCR coefficient value showing the highest comparative advantage was found in Tanah Karo Regency, North Sumatra Province in the rainy season with a DRCR coefficient value of 0.323. In the meantime, the greatest PCR and DRCR coefficients were observed. They indicated the lowest

**Table 2. Private and social costs and profits of upland potato farming, 2019-2019/2020.**

| No | Regency/Province | Financial profit (IDR) | Economic profit (IDR) |
|---|---|---|---|
| 1 | Bandung, West Java | | |
| | a. Dry season | 30,375,642 | 73,807,457 |
| | b. Rainy season | 38,224,092 | 76,357,729 |
| 2 | Wonosobo, Central Java | | |
| | a. Dry season | 73,810,641 | 126,646,043 |
| | b. Rainy season | 31,150,275 | 92,122,351 |
| 3 | Pasuruan, East Java | | |
| | a. Dry season | 35,113,165 | 71,455,486 |
| | b. Rainy season | 32,290,608 | 82,131,673 |
| 4 | Tanah Karo, North Sumatera | | |
| | a. Dry season | 18,924,987 | 73,741,736 |
| | b. Rainy season | 37,650,846 | 93,394,054 |
| 5 | Solok, West Sumatera | | |
| | a. Dry season | 40,173,656 | 90,371,212 |
| | b. Rainy season | 29,039,502 | 65,759,612 |
| 6 | Kerinci, Jambi | | |
| | a. Dry season | 41,532,849 | 92,301,180 |
| | b. Rainy season | 23,597,518 | 66,388,379 |

Source: Primary data, 2020 (processed).

https://doi.org/10.1371/journal.pone.0263633.t002

competitiveness, i.e., in Bandung Regency in the dry season with a PCR coefficient value of 0.713 and the DRCR coefficient value at the same location was DRCR 0.400. The PCR and DRCR coefficient values for potato farming by location and growing season are presented in Table 3.

**Table 3. PCR and DRCR coefficient values for dry upland potato farming in the study locations, 2019-2019/2020.**

| No. | Regency/Province | PCR | DRCR |
|---|---|---|---|
| 1 | Bandung, West Java | | |
| | a. Dry season | 0.713 | 0.499 |
| | b. Rainy season | 0.663 | 0.483 |
| 2 | Wonosobo, Central Java | | |
| | a. Dry season | 0.462 | 0.319 |
| | b. Rainy season | 0.664 | 0.382 |
| 3 | Pasuruan, East Java | | |
| | a. Dry season | 0.597 | 0.415 |
| | b. Rainy season | 0.576 | 0.341 |
| 4 | Tanah Karo, North Sumatera | | |
| | a. Dry season | 0.705 | 0.372 |
| | b. Rainy season | 0.549 | 0.323 |
| 5 | Solok, West Sumatera | | |
| | a. Dry season | 0.606 | 0.385 |
| | b. Rainy season | 0.576 | 0.462 |
| 6 | Kerinci, Jambi | | |
| | a. Dry season | 0.542 | 0.340 |
| | b. Rainy season | 0.664 | 0.401 |

Source: Primary data, 2020 (processed).

https://doi.org/10.1371/journal.pone.0263633.t003

### 4.3. Government policies impacts analysis

Impacts of government policies on the competitive performance of potato farming system can be both beneficial and harmful to farmers as the producers. There are two measures from the results of the PAM indicator analysis First, it is absolute measures consisting of output transfer (OT), input transfer (IT), factor transfer (FT) and net transfer (NT). Second, it is relative measures consisting of nominal protection coefficient on output (NPCO), nominal protection coefficient on input (NPCI), effective protection coefficient (EPC), profitability coefficient (PC) and subsidy ratio to producer (SRP).

**4.3.1. Government policy impacts on output.** The impact of government policies on potato output in research locations in Indonesia can be observed from the value of the output transfer (OT) and the nominal protection coefficient on output (NPCO). Several government policies potentially affecting potato output are trade policies, subsidies, export taxes, import tariffs, value added taxes (VAT) and supporting policies, such as irrigation infrastructure, farming roads, as well as post-harvest and product marketing.

Table 5 shows the findings from the examination of PAM indicators including output transfer (OT) and net present value (NPCO) for potato farming in Indonesian production areas. The results of the analysis showed in dry and rainy seasons, the OT value was negative and NPCO <1. The highest coefficients were found in Bandung Regency, i.e., 0.876 in rainy season and 0.865 in dry season. Meanwhile, the lowest NPCO coefficients for potato farming was found in Tanah Karo Regency, i.e., 0.748 in dry season and 0.772 in rainy season. The impact of government policies on the output of potato farmers in production areas in Indonesia was a loss or disincentive to production. It showed that in terms of output, potato farmers in Indonesia experienced a production disincentive because they accepted lower selling price than if market mechanism ran perfectly. The low selling price received by potato farmers could be due to the inefficient market chains. Increased efficiency of the market chain needs to be done to increase the selling price of potatoes at the farmer level. Increased selling prices will increase farmers' profits and incentive for producing potato commodities. The efficient market chain selection affects potato grower's profit rate [75]. The magnitude of the OT value and the NPCO coefficient value of potato farming according to location and growing season are presented in Table 4.

**4.3.2. Government policy impacts on inputs.** The impacts of government policy on tradable inputs on potato farming in production areas was indicated by the value of input transfer (IT) and nominal protection coefficient on input (NPCI). Meanwhile, the impact of government policies on domestic factors was indicated by the value of the transfer factor (FT).

Government policies affecting tradable inputs and domestic factors may consist of trade policies (export taxes and import tariffs), input subsidies, interest rate subsidies, regional minimum wages (UMR), and value added taxes (VAT). Meanwhile, other forms of divergence could be due to market distortions, such as market mechanisms not running competitively and market failure. Transfer of tradable inputs is the differences of tradable input costs at private prices and tradable input costs at social prices. Nominal protection coefficient on input (NPCI) is an input transfer indicator which is the ratio between tradable input costs calculated based on private prices and tradable input costs calculated on social prices. Values of IT, NPCI and FT in potato farming by location and growing season are presented in Table 5.

Results of the input transfer indicator for potato farming in dry highland production were positive for all seasons. Likewise, the NPCI coefficient values were > 1 in all research locations in dry and rainy seasons both in Java and Outside Java. Positive value of IT and NPCI value which is more than 1 indicate that the farmers experience disincentives on tradable input side because they are required to pay greater costs for production inputs than they might in a fully

**Table 4. Transfer output value and nominal protection coefficient on potato farming output at the research site, 2019-2019/2020.**

| No | Regency/Province | Output Transfer/OT (IDR) | Nominal Protection Coefficient on Output (NPCO) |
|---|---|---|---|
| 1 | Bandung, West Java | | |
| | a. Dry season | -30,709,200 | 0.865 |
| | b. Rainy season | -29,030,628 | 0.876 |
| 2 | Wonosobo, Central Java | | |
| | a. Dry season | -38,376,000 | 0.844 |
| | b. Rainy season | -42,612,600 | 0.800 |
| 3 | Pasuruan, East Java | | |
| | a. Dry season | -31,609,500 | 0.827 |
| | b. Rainy season | -41,677,650 | 0.774 |
| 4 | Tanah Karo, North Sumatera | | |
| | a. Dry season | -43,956,000 | 0.748 |
| | b. Rainy season | -44,623,800 | 0.772 |
| 5 | Solok, West Sumatera | | |
| | a. Dry season | -34,711,680 | 0.829 |
| | b. Rainy season | -24,846,080 | 0.861 |
| 6 | Kerinci, Jambi | | |
| | a. Dry season | -36,888,000 | 0.822 |
| | b. Rainy season | -26,175,000 | 0.854 |

Source: Primary data, 2020 (processed).

https://doi.org/10.1371/journal.pone.0263633.t004

**Table 5. Value of IT, NPCI, and FT of potato farming in dry upland in the research locations, 2019-2019/2020.**

| No | Regency/Province | Input Transfer/IT (IDR) | Nominal Protection Coefficient on Input/NPCI | Factor Transfer (IDR) |
|---|---|---|---|---|
| 1 | Bandung, West Java | | | |
| | a. Dry season | 10,815,541 | 1.135 | 1,907,074 |
| | b. Rainy season | 5,192,273 | 1.060 | 3,910,736 |
| 2 | Wonosobo, Central Java | | | |
| | a. Dry season | 10,512,400 | 1.174 | 3,947,002 |
| | b. Rainy season | 13,745,735 | 1.215 | 4,613,741 |
| 3 | Pasuruan, East Java | | | |
| | a. Dry season | 3,411,300 | 1.056 | 1,321,522 |
| | b. Rainy season | 6,829,440 | 1.115 | 1,333,974 |
| 4 | Tanah Karo, North Sumatera | | | |
| | a. Dry season | 9,457,665 | 1.167 | 1,403,084 |
| | b. Rainy season | 10,002,898 | 1.173 | 1,116,511 |
| 5 | Solok, West Sumatera | | | |
| | a. Dry season | 10,302,175 | 1.182 | 5,183,700 |
| | b. Rainy season | 7,939,999 | 1.141 | 3,934,031 |
| 6 | Kerinci, Jambi | | | |
| | a. Dry season | 12,223,200 | 1.180 | 1,657,132 |
| | b. Rainy season | 14,404,084 | 1.211 | 2,211,778 |

Source: Primary data, 2020 (processed).

https://doi.org/10.1371/journal.pone.0263633.t005

competitive market system. The NPCI with the highest value, i.e., 1.215 in rainy season, was found in Wonosobo Regency and the lowest value of NPCI, i.e., 1.056 in dry season, was discovered in Pasuruan Regency. In general, on the tradable input side, there are government policies detrimental to potato farmers because the farmers are forced to pay greater costs for tradable inputs than they should under competitive market conditions. These findings are related to study from [76] which shows government subsidies to fertiliser prices have an impact on increasing planting area and agricultural commodity yields. Market distortion is potentially due to import tariffs and value added taxes, whereas government subsidy to some agricultural commodities is abolished. Thus, farmers' access to subsidised fertilisers is also limited, especially on dry land. In addition, empirically the price of non-subsidised chemical fertilisers is much higher than those subsidised as well as the highest retail price. Compared to developed countries such as Denmark and South Korea which still receive abundant subsidies to be competitive in the global markets [76], this finding is surprising as Indonesia is one of developing countries which farmers should still receive subsidies from government. Moreover, input subsidies should be managed carefully to reach the right target. The possible strategy is empowering the existing cooperatives, not only providing subsided fertiliser but also coordinating various activities from supplying and servicing to increase farming efficiency [77].

The same phenomenon was the factor transfer in where the positive numbers were obtained in all research locations. The largest transfer factor was that in Solok Regency, West Sumatra Regency, i.e., IDR 5,183,700/ha/season in the dry season. Meanwhile, the lowest FT value was found in Tanah Karo Regency, North Sumatra Province, i.e., IDR 1,116,511/ha/season during the rainy season. The results of this analysis indicate that there are government's interventions resulting in market distortions observed by domestic variables detrimental to potato farmers. The farmers must pay higher domestic factor price than the market price. Price discrepancies of domestic factor costs between private and social prices were due to capital interest.

**4.3.3. Government policy impacts on inputs and output.** Government policy impacts on the input and output as a whole in potato production areas in Indonesia were shown by the values of Net Transfer (NT), Effective Protection Coefficient (EPC), Profitability Coefficient (PC) and Subsidy Ratio to Producer (SRP). The analysis results are depicted in Table 6.

Results of the analysis of the effective protection coefficient (EPC) of potato farming in research locations were positive with a magnitude $> 0$. The highest positive EPC value was found in Bandung Regency in the rainy season at 0.865 and in the dry season at 0.768. Meanwhile, the lowest positive EPC value was found in Tanah Karo Regency with an EPC coefficient value of 0.545 in the dry season. Policies enacted by governments or distortions in the markets affecting potato farming in general function as disincentives to growers. In most of the research locations, potato producers were not adequately protected from government policies, and they experienced disincentives in production. This is one of the reasons why Indonesia is still as a net potato importing country regardless its competitiveness. If this condition is not well anticipated, the volume of imported potatoes will continue to increase as the population and income grow.

Overall, net transfer (NT) in Indonesian potato production hubs is negative. Tanah Karo Regency, North Sumatra, has the highest negative NT value at MH of –60,972,076/ha/season, while the smallest negative NT value was found in Pasuruan Regency in the dry season of Rp -36,342,322/ha/season. Government policies or market distortions on tradable inputs and domestic factors and outputs on the potato farming system as a whole are detrimental to potato farmers. In this context, the farmers as the potato producers provide large transfers to consumer groups.

**Table 6. NT, PC, EPC and SRP values of potato farming at the research locations, 2019-2019/2020.**

| No | Regency/Province | Effective Protection Coefficient (EPC) | Net Transfer/NT (IDR) | Profitability Coefficient (PC) | Ratio Subsidy to Producer (SRP) |
|---|---|---|---|---|---|
| 1 | Bandung, West Java | | | | |
| | a. Dry season | 0.865 | -43,431,814 | 0.412 | -0.191 |
| | b. Rainy season | 0.768 | -38,133,637 | 0.501 | -0.163 |
| 2 | Wonosobo, Central Java | | | | |
| | a. Dry season | 0.737 | -52,835,402 | 0.583 | -0.214 |
| | b. Rainy season | 0.622 | -60,972,076 | 0.338 | -0.286 |
| 3 | Pasuruan, East Java | | | | |
| | a. Dry season | 0.713 | -36,342,322 | 0.491 | -0.199 |
| | b. Rainy season | 0.611 | -49,841,064 | 0.393 | -0.271 |
| 4 | Tanah Karo, North Sumatera | | | | |
| | a. Dry season | 0.545 | -54,816,749 | 0.257 | -0.315 |
| | b. Rainy season | 0.604 | -55,743,208 | 0.403 | -0.285 |
| 5 | Solok, West Sumatera | | | | |
| | a. Dry season | 0.693 | -50,197,555 | 0.445 | -0.247 |
| | b. Rainy season | 0.732 | -36,720,110 | 0.442 | -0.205 |
| 6 | Kerinci, Jambi | | | | |
| | a. Dry season | 0.649 | -50,768,332 | 0.450 | -0.245 |
| | b. Rainy season | 0.634 | -42,790,862 | 0.355 | -0.239 |

Source: Primary data, 2020 (processed).

https://doi.org/10.1371/journal.pone.0263633.t006

The results of the profitability coefficient (PC) analysis were mostly positive, but most were less than 1. In most research locations, the PC coefficient value is positive $< 1$ or 0.257–0.583, indicating that potato farmers' profits were smaller than they should get if the market mechanism worked perfectly. The greatest PC value was discovered during the rainy season in upland potato growing in Wonosobo Regency, at 0.583. Nonetheless, the PC value with the lowest was found in upland land in Tanah Karo Regency at 0.257 in Tanah Karo Regency during the dry season. Government policies or market distortions that occur in potato farming as a whole are detrimental to farmers as producers. This means that most potato farmers experience disincentives because they received lower profits than in perfectly competitive market mechanism.

Most of the subsidy ratio to producer (SRP) were negative, except in Bandung and Wonosobo Regencies in both dry and rainy seasons, and Kerinci Regency in the rainy season. The negative SRP coefficient values indicate that the potato farmers do not receive subsidies and are even burdened with taxes. The highest SRP value was found in in Bandung Regency in the rainy season (0.238), while the lowest PC value was found in Kediri Regency, i.e.-0.285, during the rainy season. Policy decisions made by the government or market distortion taking place in potato production are detrimental to farmers as producers. It indicates that, compared to a competitive market system, most potato producers cope with disincentive to produce potatoes.

## 5. Conclusion

The performance of the potato farming system in production areas in Indonesia has both financial (private) and economic (social) profitability. The highest level of financial profitability was received by farmers in Wonosobo Regency in the dry season, Central Java Province,

which was IDR 73,810,641/ha/season and economically in the same location and season is IDR 126,646,043/ha/season, meanwhile the lowest financial profit was found in Tanah Karo Regency in the dry season Rp 18,924,987/ha/season. Overall, the economic benefits received by potato farmers are greater than the financial benefits. This finding shows that potato farmers in production areas in Indonesia experience a disincentive in increasing potato production because they get a much lower profit compared to a perfectly competitive market mechanism. Despite having a comparative advantage, this finding may explain one of the reasons that Indonesia has become a net importer of potatoes. The important policy implication of this finding is that government policies should eliminate various market distortions to encourage market mechanisms to run market properly to increase the comparative advantage into competitive advantage.

Results of the comparative and competitive advantages analyses of potato farming systems in Indonesia show that it is competitive with a PCR coefficient of 1 and a DRCR coefficient of 1. The largest competitive advantage was discovered in Wonosobo Regency during the dry season with a PCR value of 0.462 and the lowest PCR was found in Bandung Regency, West Java Province, during the rainy season was 0.713. It indicates that to produce one unit of added value at private prices, the cost of using domestic resources is less than one unit. Meanwhile, from the perspective of comparative advantage the highest was found in Tanah Karo Regency during the rainy season with a DRCR of 0.323 while the lowest was found in Bandung Regency, West Java Province, in the dry season with a DRCR value of 0.499. This implies that to produce one unit of value added at social prices, it requires the use of domestic resource costs of less than one unit. In other words, to save one unit of scarce foreign exchange, the cost of using domestic resources is less than one unit. The implication for Indonesia from the perspective of efficient use of domestic resources is more profitable to increase domestic potato production than to import this commodity from the global market. These issues could be carried out through providing quality seed of improved varieties, improving cultivation technology by applying balanced fertilisers (organic and inorganic fertilisers), integrated pest and disease management, enhancing harvest and post-harvest technology, and constructing dry land agricultural infrastructure in the highlands where potato is generally grown in Indonesia, especially farming roads and irrigation networks.

The impact of government policies on tradable inputs and factor transfer gave a positive value, and the NPCI value was $>1$. This means that the impact of government policies (market distortion) on tradable inputs and domestic factors is a disincentive to the potato farming system because farmers have to pay higher tradable inputs and factor transfer than they should. More details of depth interview indicate these are caused by input prices (seeds, fertilisers, pesticides, and interest rates) that are more expensive if the market is in perfect competition. This is potentially because of input of potato seeds, non-subsidised fertilisers, and pesticides, as well as interest rates. OT values in all study sites were positive and NPCO$>1$. This means that farmers as producers get a disincentive to increase potato production indicated by lower output selling price due to non-competitive market mechanism. Simultaneously, government policies in the field of inputs and outputs in the potato farming system are detrimental to potato farmers in Indonesia because farmers do not get protection, but disincentives. Government should eliminate market distortions in the input market through improving market structure of production inputs by reducing or eliminating import duties on raw materials, encouraging competitive and healthy competition in the input market, as well as attracting investment, both domestic investment and foreign investment in the horticultural input industry. Meanwhile, the output market encourages the mechanism of the potato market competitively, improves potato-based processing industry or product down streaming, as well as expanses market and segmentations.

This study suggests that the government should develop an efficient potato farming system with an integrated agribusiness area approach in order to achieve the highest efficiency. Providing agricultural production inputs, cultivation technology, post-harvest technology, yield processing technology, consolidating farmer institutions, strengthening financing institutions, as well as agricultural product markets, and developing integrated horticultural area management institutions could be alternatives to enhancing efficiency. The development of efficient logistics and distribution systems and smooth market information services is believed to increase the competitiveness of Indonesian potatoes in the domestic and global markets. To increase comparative advantage, it is necessary to manage natural resources in an integrated and sustainable manner, support agricultural infrastructure (farming roads, irrigation infrastructure) and post-harvest infrastructure and logistics (packing houses, public roads, and central warehousing and distribution systems), the use of certified quality potato seed, fertiliser application according to the recommendation package, the provision of organic fertiliser, agricultural mechanisation specific to dry land, as well as wider market access. In addition, availability of packing houses, cold storage, and refrigerated transportation modes need to be developed at the regional level. Policy on increasing the availability of quality potato seeds, eliminating distortions in the input and output markets, access to credit sources with competitive interest rates, and encouraging the operation of mechanisms for both input and output of potatoes in a more competitive and equitable manner are necessary. Lastly, policy makers may pay attention to the importance of increasing market access is not only in the domestic market but more importantly access to the global market through bilateral, multilateral and inter-regional trade cooperation.

## Supporting information

**S1 File.**
(DOCX)

## Acknowledgments

The authors gratefully acknowledge Professor Eric A. Monke and Prof. Scott Pearson through their book "The Policy Analysis Matrix for Agricultural Development" and Prof. Scott Pearson, Carl Gotsch, and Syaiful Bahri through their book entitled "Applications of the Policy Analysis Matrix in Indonesia Agriculture." The authors also acknowledge the assistance of the local staff in the Agricultural Services districts and other government authorities in conducting this research. Despite their busy working schedules, the farmers' group and farmer respondents' cooperation during the interviews and field surveys is highly appreciated.

## Author Contributions

**Conceptualization:** Saptana, Bambang Sayaka, Yusuf, Muhammad Prama Yufdy.

**Data curation:** Saptana, Atika Dyah Perwita, Endro Gunawan, Syahrul Ganda Sukmaya, Sumaryanto.

**Formal analysis:** Saptana, Bambang Sayaka, Atika Dian Pitaloka.

**Investigation:** Saptana, Apri Laila Sayekti, Nur Qomariah Hayati, Sudi Mardianto.

**Methodology:** Saptana, Apri Laila Sayekti, Sumaryanto.

**Supervision:** Saptana, Muhammad Prama Yufdy.

**Validation:** Atika Dyah Perwita, Bambang Sayaka, Nur Qomariah Hayati, Sudi Mardianto.

**Writing – original draft:** Saptana, Apri Laila Sayekti, Atika Dyah Perwita, Bambang Sayaka, Endro Gunawan, Syahrul Ganda Sukmaya.

**Writing – review & editing:** Endro Gunawan, Yusuf, Sumaryanto, Muhammad Prama Yufdy, Atika Dian Pitaloka.

## References

1. [WTO] World Trade Organization. Global value chain development report 2019: technological innovation, supply chain trade, and workers in the globalized world. 2019.
2. World Bank. World development report 2020. Trading for development in the age of global value chains. 2020. https://doi.org/10.1016/j.gaceta.2020.05.010 PMID: 32674862
3. Michaličková M, Krupova Z, Polak P, Hetenyi L, and Krupa E. Development of competitiveness and its determinants in Slovak dairy farms. Agricultural economics, 2012;(2):82–88. https://doi.org/10.17221/76/2013-AGRICECON.
4. Siudek T, and Zawojska A. Competitiveness in the economic concepts, theories, and empirical research. Acta Scientiarum Polonorum. Oeconomia. 2014; 13(1).
5. Parzonko A, and Bórawski P. Competitiveness of Polish dairy farms in the European Union. Agricultural Economics. 2020; 66(4): pp.168–174. https://doi.org/10.17221/254/2019-AGRICECON.
6. Gereffi G, and Stark KF. Global value chain analysis: a Primer. Center on globalization, governances, and competitiveness. The Social Science Research Institute. 2016.
7. [UNIDO] United Nations Industrial Development Organization. Global value chains and development. New York (US): UNIDO Publishing. 2015.
8. [WTO] World Trade Organization. Global value chain development report 2017: measuring and analyzing the impact of GVCs on economic development. Washington DC (US): World Bank Publishing. 2017.
9. World Bank. World development report 2020: trading for development in the age of global value chains. Washington DC (US): World Bank Publishing. 2019.
10. Moon W. Are there dynamic productivity gains from agricultural trade?". China Agricultural Economic Review. 2021. https://doi.org/10.1108/CAER-02-2021-0030.
11. Gultom D. Regional comprehensive economic partnership (RCEP): how Indonesia can maximize the opportunities an insider's perspective. Policy Brief No.6. Center for Indonesian Policy Studies (CIPS). 2020.
12. Friyatno S, & Saptana. Kinerja agribisnis komoditas pertanian: kemampuan penciptaan output, nilai tambah dan keterkaitan antar sektor (analisis komparasi I-O tahun 2005 dan 2010). Jurnal Manajemen & Agribisnis. 2017; 14(3):250–263.
13. Ingot SR, Laksani DD. Indonesia global value chain participation in regional comprehensive economic partnership (RCEP). Advance in Economic and Management Research. 2019; 98: 159–163.
14. Kaplinsy R. Globalization, and unequalization: what can be learned from value change analysis. Journal of Development Studies. 2010; 37(2): 117–46.
15. Felice G, and Tajoli L. Trade balances and global value chains: Is there a link?. *Structural Change and Economic Dynamics*. 2021; 59: 228–246. https://doi.org/10.1016/j.strueco.2021.08.013.
16. Firmansyah, Widodo W, Karsinah, Oktavia S. Export Performance and Competitiveness of Indonesian Food Commodities. Jurnal Ekonomi dan Kebijakan 2017; 10(2):289–310. https://doi.org/10.1002/aur.1651 PMID: 27333365
17. Statistics Indonesia. Statistics of Horticulture 2020. Jakarta: Statistics Indonesia. 2021.
18. Hameed TS, Sawicka B. Production and marketing problems of potato growers. International Journal of Current Research. 2017; 9: 49729–49732, May 2017.
19. Hassan SZ., Jajja MSS, and Foster G. Bringing more value to small farmers: a study of potato farmers in Pakistan. Management Decision. 2021. Vol. 59 No. 4, pp. 829–857. https://doi.org/10.1108/MD-12-2018-1392.
20. Balassa B. The theory of economic integration. Massachusetts (US): Homewood Illinois: RD Irwin Inc. 1965.
21. Balassa B, Noland M. "Revealed" Comparative Advantage in Japan and the United States. Journal of International Economic Integration 4(2) Autumn. 1989; 8–22.

22. Ningsih I, Kurniawan W. Daya saing dinamis produk pertanian Indonesia di ASEAN. Jurnal Ekonomi Kuantitatif Terapan. 2016; 9(2):117–125.

23. David Gaytán Francisco Benita. On the competitiveness of Mexico's dry chili production. EP 2014 (61) 2 (307–317).

24. Ferto I, Hubbard LJ. Revealed comparative advantage and competitiveness in Hungarian agri-food sectors. The World Economic. 2003; 26(2):247–259.

25. Kumar NR, Rai M. Performance, competitiveness and determinants of tomato export from India. Agricultural Economics Research Review. 2007; 20: 551–562.

26. Laursen K. Revealed comparative advantage and the alternatives as measures of international specialization. Working Paper No 98–30. Danish Research Unit for Industrial Dynamics (DRUID). 1998.

27. Laursen K. Revealed comparative advantage and the alternatives as measures of international specialization. Eurasian Business Review.2015; 5(1): 99–115.

28. Athukorala P. Post-Crisis Export Performance: The Indonesian Experience in Regional Perspective, Bulletin of Indonesian Economic Studies. 2006; 42(2), 177–211.

29. Abdullahi NM, Zhang Q, Shahriar S, Kea S, Huo X. Relative export competitiveness of the Nigerian cocoa industry. Competitiveness Review: An International Business Journal. 2021. https://doi.org/10.1108/CR-03-2021-0036.

30. Monke EA, dan Pearson SR. The Policy Analysis Matrix for Agricultural Development. Cornel University Press, Ithaca, and London. 1989.

31. Monke, Eric A, and Scott R. Pearson. The Policy Analysis Matrix for Agricultural Development. Cornel University Press. Ithaca and London. 1995.

32. Pearson PS, Bahri& Gostch C. Is Rice Probability in Indonesia Still Profitable? FPSA Working Paper, February 2005. California: Stanford University. 2005.

33. Kozár L, Vasa L & Neszmélyi GI2021. The Opportunities and Challenges of Combined Use of Futures Commodity Exchange and Public Warehouse in the Crop Sector of Vietnam. European Research Studies. Vol. 24, pp. 1260–1286.

34. Van Hoang V. Investigating the agricultural competitiveness of ASEAN countries. Journal of Economic Studies. 2020. https://doi.org/10.1108/JES-10-2018-0366.

35. Kusuma RL, Firdaus M. Daya saing dan faktor yang memengaruhi volume ekspor sayuran Indonesia terhadap negara tujuan utama. J Manaj Agribisnis. 2015; 12(3):226–36. https://doi.org/10.17358/jma.12.3.226.

36. Fauziah DS. Analisis Daya Saing dan Faktor-Faktor Yang Mempengaruhi Tingkat Ekspor Kentang di Singapura dan Malaysia. Skripsi, Program Studi Pembangunan Jurusan Ilmu Ekonomi dan Studi Pembangunan Fakultas Ekonomi dan Bisnis Universitas Jember. 2019.

37. Daryanto A. Posisi dan daya saing pertanian Indonesia dan upaya peningkatannya. Makalah disampaikan pada Seminar Nasional PSEKP: Peningkatan daya saing agribisnis berorientasi kesejahteraan petani, 2009 Okt 14; Bogor: (ID).

38. Saptana. Tinjauan konseptual mikro-makro daya saing dan strategi pembangunan pertanian. Jurnal Forum Penelitian Agro Ekonomi. 2010; 28(1):1–18.

39. Rambe P. and Khaola P. The impact of innovation on agribusiness competitiveness: the mediating role of technology transfer and productivity. European Journal of Innovation Management. 2021. https://doi.org/10.1108/EJIM-05-2020-0180.

40. Gál T, Nagy L, Dávid L, Vasa L & Balogh P. Technology planning system as a decision support tool for dairy farms in Hungary. 2013. Acta Polytechnica Hungarica. Vol. 10, no. 8, pp. 231–244.

41. Kart MCO, Abay CF, Gungor S, Ozer Z. Seed Supply and Seed Preferences of Potato Farmers: Nigde Central and Izmir Odemis Provinces, Scientific Papers Series Management, Economic Engineering in Agriculture and Rural Development. 2017; 17: 239–249.

42. Grupe C, Rose A. China, India, and the Socioeconomic Determinants of Their Competitiveness. Journal of Economics Research International, Article ID 860425. 2010;1–15. 2010.

43. Porter ME. Competitive of nations. New York: The Free Press. 1990.

44. Smith M. Issues of competitiveness and regional growth in relation to transport infrastructure investment: A literature review on assessment methodology; 2013. Available at: http://www.i-c-eu.eu/deliverables/I-C-EU_WP1_D1.1.pdf (Accessed 14/10/2014).

45. Sachitra V. and Chong SC. Resources, capabilities and competitive advantage of minor export crops farms in Sri Lanka: An empirical investigation. Competitiveness Review. 2018. Vol. 28 No. 5, pp. 478–502. https://doi.org/10.1108/CR-01-2017-0004.

46. Newbert SL. Value, rareness, competitive advantage, and performance: A conceptual-level empirical investigation of the resource-based view of the firm. Strategic Management Journal. 2008; 29: 745–768.

47. Manalu DST, Suharno, dan Tinaprilla N. Daya Saing Komoditas Kentang di Kabupaten Banjarnegara, Jawa Tengah Pendekatan *Policy Analysis Matrix* (PAM). Jurnal Sains Terapan. 2014; 1: 1–14.

48. Selvia M, Djuwendah E. Strategi pengembangan kentang (*solanum tuberosum l.)* pada Kelompok Tani Palintang Jaya, Desa Cipanjalu Kecamatan Cilengkrang, Kabupaten Bandung. Agricore. 2018; 3: 548–561.

49. Kiloes AM, Sayekti AL, Anwarudin Syah MJ. Potato competitiveness evaluation in production center of Pangalengan, Bandung Regency. J. Hort. 2015: 25(1):88–96.

50. Huo D. Impact of country-level factors on export competitiveness of agriculture industry from emerging markets. Competitiveness Review. 2014. Vol. 24 No. 5, pp. 393–413. https://doi.org/10.1108/CR-01-2012-0002.

51. Savitri PDL, Budhi MKS. Analisis pengaruh produksi kentang, inflasi, dan nilai tukar terhadap ekspor kentang Indonesia periode 1993–2013. E- Jurnal EP Unud, 2015: 4(7): 763–775.

52. Saini I, Kaushik P, Al-Huqail AA, KhanF, and Siddiqui MH. Effect of the diverse combinations of useful microbes and chemical fertilizers on important traits of potato. *Saudi Journal of Biological Sciences*, 2021: 28(5): 2641–2648. https://doi.org/10.1016/j.sjbs.2021.02.070 PMID: 34025148

53. Alfalluji SJI. The comparative advantage, competitiveness, and added value of potato crop of Baghdad province for the year 2018. Plant Archives; 2019; 19:1645–1655.

54. Gebru H, Mohammed A, Dechassa N, Belew D. Assessment of production practices of smallholder potato (*Solanum tuberosum* L.) farmers in Wolaita zone, southern Ethiopia. *Agric & Food Security*. 2017; 6:31. https://doi.org/10.1186/s40066-017-0106-8.

55. Kempenaar C, Been T, Booij J, van Evert F, Michielsen JM, Kocks C. Advances in Variable Rate Technology Application in Potato in The Netherlands. Potato Research. 2017; 60:295–305. https://doi.org/10.1007/s11540-018-9357-4 PMID: 30956286

56. Ahmed MEM. Competitiveness Indicators of Egyptian Potatoes in the Global Market. Journal of Applied Sciences Research. 2019: 3632–3637.

57. Halterman D, Guenthner J, Collinge S, Butler N, Douches D. Biotech Potatoes in the 21st Century: 20 Years Since the First Biotech Potato. Am. J. Potato Res. 2016; 93:1–20. https://doi.org/10.1007/s12230-015-9485-1.

58. McFadden JR, and Huffman WE. Consumer valuation of information about food safety achieved using biotechnology: Evidence from new potato products. *Food Policy*. 2017; 69, pp.82–96. https://doi.org/10.1016/j.foodpol.2017.03.002.

59. Gitari HI, Nyawade SO, Kamau S, Karanja NN, Gachene C., Raza MA, et al. Revisiting intercropping indices with respect to potato-legume intercropping systems. Field Crops Research, 2020; 258; p.107957. https://doi.org/10.1016/j.fcr.2020.107957.

60. Vasa L. Behaviour patterns of farm-managing households after the restructuring of agriculture–a socio-economic analysis. Journal of Central European Agriculture. 2002, 3(4):313–320.

61. Cieslik K, Cecchi F, Damtew EA, Tafesse S, Struik PC, Lemaga B, et al. The role of ICT in collective management of public bads: The case of potato late blight in Ethiopia. World Development. 2021; 140; p.105366. https://doi.org/10.1016/j.worlddev.2020.105366.

62. Lindert P H, dan Kindleberger ChP, Ekonomi Internasional (Alih Bahasa Burhanuddin Abdullah) Edisi Kedelapan. Penerbit Erlangga Jakarta. 1993.

63. Krugman PR, Dan Obstfeld M. Ekonomi Internasional Teori dan Kebijakan. Edisi Kedua. Universitas Indonesia dan HasperCollins Publishers. 1994.

64. Purnamawati Astuti, Sri F. Dasar-dasar ekspor impor. UPP STIM YKPN. Yogyakarta. 2013.

65. Salvatore D. Ekonomi Internasional. Erlangga. Jakarta. 1994.

66. Heckscher EF. Utrikshandelns verkan pa inkomstfoerdelningen, Ekonomist Tradskrift, 21, Del 2, 1–32 (The effect of foreign trade on the distribution of income, in Ellis H. S. and Metzler L. A. eds., Readings in the Theory of International Trade, 1949, Philadelphia: Blakiston, 272–300. 1919.

67. Oliinyk O, Bilan Y, Mishchuk H, Akimov O, & Vasa L. 2021. The Impact of migration of highly skilled workers on the country's competitiveness and economic growth. Montenegrin Journal of Economics. 2021, 17(3): 7–13.

68. Ohlin B. Interregional and International Trade, Cambridge, Mass.; Harvard University Press. 1933.

69. Esterhuizen Dirk JV, Royen and Luc D'Haese. An Evaluation of The Competitiveness Sector in South Africa. Advanced in Competitiveness Research. 2008; 16: 31–46.

70. Tufail S. Comparative Advantage of Major Crops in Punjab-Pakistan: An Application of Policy Analysis Matrix. M. Phil. A thesis submitted to the Federal Urdu University of Arts, Science and Technology, Islamabad. 2014.

71. Salam A, Tufail S. Competitiveness and Comparative Advantage of Important Food and Industrial Crops in Punjab: Application of Policy Analysis Matrix. Journal of International Agricultural Trade and Development. 2016; 10:81–94.

72. Chowdhury SMZI. Construction of a Policy Analysis Matrix (PAM) for Fruits and Vegetables Export Process in Bangladesh. Turkish Research Journal of Academic Social Science. 2020; 3(1): 11–21.

73. Gittinger JP. Analisis Ekonomi Proyek-Proyek Pertanian Edisi 1986 (Terjemahan). Universitas Indonesia Press. Jakarta. 1986.

74. Saptana, Gunawan E, Perwita AD, Sukamaya SG, Darwis V, Ariningsih E, Ashari. The competitiveness analysis of shallot in Indonesia: A Policy Analysis Matrix. PloS ONE. 2021; 16(9):e0256832. https://doi.org/10.1371/journal.pone.0256832 PMID: 34478456

75. Kyomugisha H., Mugisha J. and Sebatta C. Potential determinants of profits and market efficiency of potato market chains in Uganda. Journal of Agribusiness in Developing and Emerging Economies. 2017. Vol. 7 No. 1, pp. 52–68. https://doi.org/10.1108/JADEE-06-2015-0031.

76. Hodjo M, Ram A, Blayney D, Nakelse T. Impacts of price, weather and policy changes on maize and rice farming in Togo. African Journal of Economic and Management Studies. 2021. Vol. 12 No. 3, pp. 357–380. https://doi.org/10.1108/AJEMS-09-2020-0427.

77. Neszmelyi GI. The Main Characteristics of The South Korean Agriculture. Economic and Local Aspects of Rural Development, Szent Istvan University, Budapest, Hungary, 2017:31–43.
