# Supplementary material for: Competitiveness analysis of fresh tomatoes in Indonesia: Turning comparative advantage into competitive advantage
Source: PLoS One. 2023 Nov 30;18(11):e0294980. doi: 10.1371/journal.pone.0294980 (PMC10688876; doi:10.1371/journal.pone.0294980)
Supplement: Attachment — Submitted filename: 10.1515_opag-2022-0083-CHILI.pdf [file pone.0294980.s003.pdf]

Research Article

Saptana Saptana, Ening Ariningsih, Ashari Ashari, Endro Gunawan*, Atika Dyah Perwita, Syahrul Ganda Sukmaya, Handewi Purwati Saliem, Helena Juliani Purba, Kurnia Suci Indraningsih, Atika Dian Pitaloka, Nur Qomariah Hayati

Competitiveness and impact of government policy on chili in Indonesia

https://doi.org/10.1515/opag-2022-0083
received September 26, 2021; accepted March 14, 2022

Abstract: Indonesian chili faces some problems in increasing production, added value, and competitiveness of chili products, mainly in terms of quantity, quality, and continuity. The objectives of this study are (1) to analyze the private and social profitability of chili farming, (2) to analyze the chili competitiveness from both competitive and comparative advantage perspectives, (3) to examine government policy impact on chili performance, and (4) to formulate strategies to encourage chili development in Indonesia. The results of the policy analysis matrix revealed that chili farming in Indonesia's production centers is profitable, both privately and socially. It also has competitiveness, both competitive and comparative advantages. The highest competitiveness occurs in Bandung district, West Java, with a coefficient of private cost ratio (PCR) of 0.416 and a domestic resource cost ratio (DRCR) of 0.269. Meanwhile, the lowest competitiveness occurs in Tabalong district, South Kalimantan, with a PCR coefficient of 0.857 and a DRCR of 0.556. This study also concluded that for Indonesia, it is more profitable to increase domestic chili production than importing from abroad. Strategic policies for chili development can be implemented by using hybrid seeds, complete and balanced fertilization, improving irrigation infrastructure and farming roads, increasing the capacity of farmers' resources, and expanding the objectives and market segments.

Keywords: chili, competitiveness, policy analysis matrix, Indonesia

*** Corresponding author: Endro Gunawan,** Indonesian Center for Agricultural Socio Economics and Policy Studies, Bogor, Indonesia, e-mail: endrogun007@gmail.com
Saptana Saptana, Ening Ariningsih, Ashari Ashari, Handewi Purwati Saliem, Helena Juliani Purba, Kurnia Suci Indraningsih: Indonesian Center for Agricultural Socio Economics and Policy Studies, Bogor, Indonesia
Atika Dyah Perwita: Faculty of Education and Teacher Training, Walisongo State Islamic University, Semarang, Indonesia
Syahrul Ganda Sukmaya: Faculty of Agriculture, Universitas Jenderal Soedirman, Purwokerto, Indonesia
Atika Dian Pitaloka: School of Business IPB University, Bogor, Indonesia
Nur Qomariah Hayati: Indonesian Center for Horticulture Research and Development, Bogor, Indonesia

1 Introduction

The economic world has been moving toward stronger cross-national economic relations [1]. It implies that trade liberalization is increasingly providing opportunities and challenges for all horticultural commodity business actors, including farmers. Liberalization could lead to better world agricultural prices and increased activity and rewards in the agricultural sector [2]. In the era of free trade, business competition is getting more challenging, so every supply-chain actor is required to improve the competitiveness of their products. Trade liberalization provides new business opportunities as trade barriers between countries are removed from the market demand side. However, on the other hand, liberalization can also cause severe problems if domestic products cannot compete in the global market.

From a regional trade perspective, adding value for agricultural products can be created if Indonesia is involved in regional and global value chains (GVCs) [3]. This participation is even more crucial for horticultural products, considering that, in aggregate, these products have a strong forward and backward linkage index with other industries [4,5]. In addition, the use of value chain analysis in the horticulture subsector can stimulate economic activity in the country and contribute more to even income distribution among the business actors [6].

One of the requirements to gain an advantage in the global market is to be competitive. Product competitiveness

Open Access. © 2022 Saptana *et al.*, published by De Gruyter. This work is licensed under the Creative Commons Attribution 4.0 International License.

in the export market is determined by the overall performance of the product value chain, both at the domestic and global levels [7,8]. Recently, the agricultural value chain has grown more global market-oriented. Hence, it is frequently referred to as the GVC [9–11]. Meanwhile, the core of the competitiveness of an industry or product is efficiency and productivity [12].

Some studies use several indicators to measure the competitiveness of a commodity. The revealed comparative advantage index is widely used to measure competitive advantage in the agricultural sector, especially horticultural products [13,14]. The study by Notta and Vlachvei [15] uses market share, profitability, and productivity to measure the competitive advantage of the food and beverage processing industry.

The study results related to trade in Indonesian agricultural products in the global market [16] show that plantation products have reached the maturity stage and have a comparative advantage. On the other hand, food and horticultural products are still in the introductory stage and do not yet have a comparative advantage. Based on data from the Ministry of Agriculture [17], Indonesia has succeeded in exporting chili, mainly to Malaysia and Saudi Arabia, in which 97% of the total chili exports are processed products.

The private cost ratio (PCR) and domestic resource cost ratio (DRCR) are two other indicators used when the policy analysis matrix (PAM) is used to assess competitiveness. PAM calculates private and social costs, tradable input components, and domestic factors [18,19]. PCR is a competitive advantage indicator indicating the amount of domestic resources to conserve to produce a single output unit at private prices. Farming activity has a competitive advantage if the PCR value is <1: the smaller, the more efficient. DRCR is a comparative advantage indicator that shows the amount of domestic resources that can be kept to create one foreign-exchange unit. Farming activity has a comparative advantage in the absence of government policies or perfectly competitive market circumstances if the DRCR value is <1: the smaller, the more efficient.

The PAM approach has been applied to various competitiveness studies or for formulating agricultural policies. Pearson et al. [19] explore a multi-case study to evaluate Indonesian agriculture policy. Such an approach is also used to measure the competitiveness of corn farming in a marginal area [20], robusta coffee [21], Pronojiwo snake fruit in East Java province [22], and investigate rice plant [23] and tobacco [24] input subsidy policies. PAM is also used to assess the impact of intensifying rice production systems in Southeastern Nigeria [25] and analyze the profitability of rice farming in India [26]. Souza et al. [27] combine primary data from representative companies and secondary data to make economic and accounting evaluations of the rice production chain in Rio Grande do Sul (Brazil) and Uruguay.

It has become an everyday discourse in development theory that sometimes government intervention needs to be carried out to encourage product and economic competitiveness. Interventions in the economic system are intended to stimulate the development of specific sectors (vertical policies) or the industry as a whole (horizontal policies), otherwise known as industrial policy [28]. The literature on industrial policy argues that government intervention can help overcome market failures and provide incentives for further industrial development [29]. The PAM method is generally well able to explain the impact of government intervention on specific sectors/industries.

PAM can also be used to analyze horticultural commodities, including chili. However, it is still rare for international studies to use PAM as an analytical method to investigate the comparative and competitive advantages of chili. The chili study in India [30] did not use PAM but an indicator of export performance ratio and revealed symmetric comparative advantage. This study concludes that India is not competitive in exporting both dried chilies and green chilies.

In the context of Indonesia, a study on chili competitiveness (comparative and competitive advantages) is necessary to provide empirical responses on chili competitiveness while also contributing to the literature on the topic. Chili is a national strategic commodity because fluctuations in chili prices affect the inflation rate. Despite the strong recommendation to improve production and its continuity [31,32], chili development still faces several challenges, including technological, socioeconomic, and policy support.

Some previous studies show that Indonesian chili has comparative and competitive advantages [33–36]. In addition to competitiveness, other studies related to chili also discuss several other aspects such as business feasibility [37,38], supply-chain/agribusiness development [32,39,40], inhibitors and drivers of chili agribusiness [41], institutions and strategies for increasing competitiveness [42], marketing channels [43], and transaction cost structure and its effect on the revenue and profit of chili farming [44].

However, those studies are still limited to one region/district and agroecosystems, such as wetland (rice field)/dryland and lowland/upland. There is no comprehensive study comparing the competitiveness of chili between provinces or between islands (Java and outside Java) and between types of agroecosystems in Indonesia. Therefore, this study is expected to fill the study gap and discuss the competitiveness of chili with broader coverage.

Some issues require to be explained to analyze the competitiveness of chili farming. (1) Does chili farming have comparative and competitive advantages in the market? (2) How is the competitiveness of chili between agroecosystems and between growing periods? (3) Does the existing competitive situation have prospects to be sustained? (4) What incentive policy options can turn their comparative advantage into a competitive advantage in the market? This study aims to (1) examine the private and social profitability of chili farming, (2) analyze the competitiveness of chili farming from both competitive and comparative advantage perspectives, (3) assess government policy impact on chili farming, and (4) formulate policy strategy to increase the competitiveness of chili.

2 Methods

2.1 Research location

This research was conducted in six districts of chili production centers in Indonesia, representing wet-lowland and dry-upland agroecosystems in the regions of Java island and outside Java island. Kediri district, East Java province; Tabalong district, South Kalimantan province; and Bantaeng district, South Sulawesi province, represented wet-lowland agroecosystem. Meanwhile, Bandung district, West Java province; Banjarnegara district, Central Java province; and Tanah Karo district, North Sumatra province, represented the dry-upland agroecosystem. The location of the study is shown in Figure 1.

2.2 Data collection

Data were collected through three approaches: (1) focus group discussion (FGD), involving the group leader (one

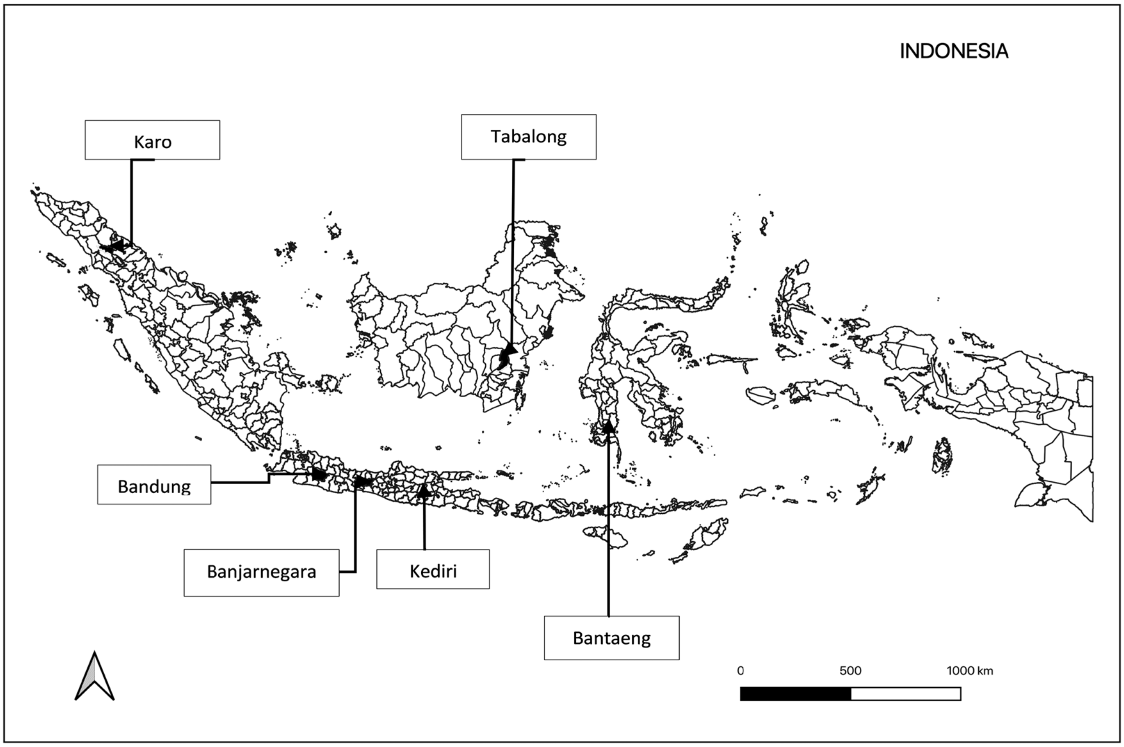

Figure 1: Location of the study.

person), group administrators (two people), farmer group members (three people); (2) a limited survey with 10–15 respondents representing chili farmers with large land tenure (30%), farmers with medium land tenure (40%), and farmers with narrow land tenure; and (3) interviews with chili supply-chain actors, i.e., village-level collectors, inter-regional traders, wholesalers, and retailers. The data collected through FGDs overview the area, farming system, cropping patterns, technology adoption rates, productivity levels, and social prices for domestic factors (labor wages, land rent, capital interest). In addition, secondary data are also used, especially the limit price as a shadow price for tradable goods.

The data collected for a limited survey (10–15 people) include the structure of farm inputs and outputs, input and output prices, and farm input and output values. The FGD and the limited survey data were used to compile a financial and economic (social) analysis of chili farming in each location. Meanwhile, interviews with traders at various levels help estimate the transportation costs for imported goods in each location.

The survey recalled data of the 2019 dry season and the 2019/2020 rainy season. The selection of respondents followed the stratified random sampling method. The analysis was done on a per hectare basis corresponding to the agroecosystem (wet-lowland and dry-upland) and planting season (rainy and dry seasons). The data collection was conducted from January to April 2020.

2.3 Methods of analysis

By using the PAM analysis, economic activity can be considered from two viewpoints, i.e., private and social [12,18,19,33,34,36,45]. Basically, PAM calculation comprises four stages: (1) the physical input–output determination of the whole farming business; (2) the estimation of shadow/social prices of inputs and outputs; (3) the breakdown of entire cost components into tradable inputs and domestic factors and calculation of the amount of financial (private) and economic (social) revenues; and (4) the calculation and analysis of 13 indicators that can be generated from PAM. Before calculating PAM, two essential issues are to be explained, i.e., the cost separation into tradable inputs and domestic factors and shadow/social price estimation of inputs and outputs.

2.3.1 Allocation of components into tradable inputs and domestic factors

PAM needs to separate the production inputs into tradable inputs (tradable goods) and domestic factors (non-tradable goods). The first type of input is input that can be transacted in global markets, and the contrary is for the second type. Tradable goods have the following characteristics: (1) goods that are currently being exported or imported; (2) goods that are alternates are strongly related to other types that are exported or imported; (3) goods other than the above and get protection from the government, which can be traded globally [18,19]. This means that if there is a shortage of these goods, they can be obtained from the world market and *vice versa*.

Two approaches can be used to distribute costs into the components of tradable inputs and domestic factors, i.e., the total approach and the direct approach [18,19]. This research uses a direct approach by assuming that all costs of tradable inputs, both imported and domestic products that can be traded globally, are valued as components of tradable inputs. This approach was chosen because of the increasing occurrence of free trade and the increased demand for tradable inputs, both imported and domestically produced goods, which the global market can influence.

Several previous studies have widely applied this direct approach [33,46]. In this article, chili as output is considered 100% tradable goods. Inputs that are assumed 100% tradable include chili seed, fertilizers (urea/ZA, SP-36/TSP, KCl/KNO3, NPK), pesticides, and plastic mulch. Meanwhile, organic fertilizers, liquid organic fertilizers, dolomites, raffia ropes, stakes, labor, land lease, and capital interest are considered 100% domestic factors.

In transportation activities, the costs incurred are allocated into tradable inputs and domestic factors based on information from some players in the trading system. The labor costs are assumed as domestic factors, whereas transportation costs are considered a component of tradable inputs. In post-harvest activities, the cost distribution into tradable inputs and domestic factors is done based on the information from chili business actors. The post-harvest costs consist of materials included in tradable inputs and labor categorized as domestic factors. In detail, the allocation of costs into the elements of tradable inputs and local factors of chili farming is presented in Table S1.

2.3.2 Determination of social price

Two price levels are specified for each input and output: the market price (private price) and the social price. The level of the market price received by farmers in the sale of output, or the level of prices paid by farmers in acquiring factors of production, is referred to as the private price. Shadow prices occur in the economy under perfect competition and equilibrium conditions. It is not easy to find a counterbalanced cost equal to the market price. It needs to adjust the prevailing market price to obtain a value close to the opportunity cost or social price. The shadow price is calculated by removing distortions caused by government policies such as subsidies, taxes, minimum wage determination, pricing controls, import tariffs, and other chili-related measures.

This study uses the border price approach for tradable goods. For exported goods, f.o.b. (free on board) prices are used, whereas for imported commodities, c.i.f. (cost insurance freight) prices are used by making various adjustments to the level at which competition occurs. Meanwhile, the opportunity costs or the average prices in each research location are used for domestic factors. This study used a conversion rate of IDR 14,141/ USD 1. In detail, the estimation results of input and output shadow prices for chili farming are presented in Tables S2 and S3.

2.3.3 Formation of PAM

The PAM calculation step comprises four stages: (1) determining the whole physical inputs and outputs of the economic activities analyzed; (2) separating all cost components into tradable inputs and domestic factors, and calculating the amount of revenue (Table S1); (3) assessing the shadow price of inputs and outputs (Tables S2 and S3); and (4) calculating and analyzing various indicators resulting from the PAM analysis, which have been presented in the analysis table in this article (Tables S4–S9).

The PAM formation is conducted after collecting all data from the farmers and chili supply-chain actors. The PAM is created using an input–output structure at the farm level and trading system actors, focusing on transportation costs. Both financial and economic profits can be acquired using this calculation. The impact of government policies on inputs, outputs, and both inputs and outputs can be determined simultaneously.

The findings of PAM analysis reveal profitability, competitiveness from the standpoints of economic efficiency (comparative advantage) and financial efficiency (competitive advantage), and government policy impact on chili farming systems. Table 1 presents the PAM table used in this analysis.

3 Results and discussion

Some economists interpret competitive advantage as the result of a combination of market distortions and comparative advantage [18,19]. Related to the concept of comparative advantage is economic feasibility, and related to the concept of competitive advantage is the financial feasibility of an activity. Gupta [47] shows how competitive and comparative advantage models complement each other in determining and maintaining a country's competitiveness in international trade and business.

3.1 Private and social costs and profits

The results of the financial (private) cost and profit analysis demonstrate that chili farming in chili-producing centers in Indonesia, both wet-lowland and dry-upland, yields good profits in both the dry and rainy seasons. Economic (social) profits are generally higher than

Table 1: Policy analysis matrix (PAM) [20]

Description	Revenues	Costs		Profits
		Tradable inputs	Domestic factors	
Private prices	A	B	C	D
Social prices	E	F	G	H
Divergences	I	J	K	L

Note: I = A − E; J = B − F; K = C − G; L = D − H.
1. Private profits (PP): D = A − (B + C).
2. Social profits (SP): H = E − (F + G).
3. Private cost ratio (PCR) = C/(A − B).
4. Domestic resource cost ratio (DRCR) = G/(E − F).
5. Output transfers (OT): I = A − E.
6. Nominal protection coefficient on tradable outputs (NPCO) = A/E.
7. Input transfers (IT): J = B − F.
8. Nominal protection coefficient on tradable inputs (NPCI) = B/F.
9. Factor transfers (FT): K = C − G.
10. Effective protection coefficient (EPC) = (A − B)/(E − F).
11. Net transfers (NT): L = D − H.
12. Profitability coefficient (PC) = D/H.
13. Subsidy ratio to producers (SRP) = L/E.

financial (private) profits, and profits during the rainy season are higher than profits during the dry season. However, even though it is profitable both privately and socially, the risks in chili agribusiness are high [48,49] because of production, market, and institutional risks, which have implications for financial risk [49]. This is alarming since the majority of chili farming was done by smallholder farmers [50].

Table 2 shows that chili farming in all research locations is profitable financially and economically, both in the dry and rainy seasons, implying that it is financially and economically feasible. Some previous studies related to chili farming attest to this claim. For instance, chili farming in Samarinda (East Kalimantan province) generates total revenue of IDR 67,500,000 and R/C = 2.39 [51], whereas in Ciamis (West Java province) provides a profit of IDR 73,940,000 and R/C = 2.51 [52]. Other studies in the different areas also support the profitability and feasibility of chili farming (including cayenne), such as in Central Sulawesi [53], East Java [38,41,54,55], West Java [56], North Sumatra [37], and South East Sulawesi provinces [57].

Chili farming in Bandung district has the highest private profit in both the dry and rainy seasons at IDR 71,460,774/ha/season and IDR 58,278,225/ha/season, respectively. Meanwhile, the lowest private profit is experienced by chili farming in the Bantaeng district in the dry season (IDR 34,829,813/ha/season) and the Banjarnegara district in the rainy season (IDR 22,348,570/ha/season).

In the dry season, the highest social or economic profits occur in Kediri district (IDR 98,425,650/ha/season), whereas in the rainy season, it occurs in Tanah Karo district (IDR 107,393,438/ha/season). Meanwhile, the lowest social profits occur in Tabalong district at IDR 19,369,121/ha/season in the dry season and IDR 13,348,843/ha/season in the rainy season. Table 2 presents the private and social profits of chili farming in research locations. In addition, the results of PAM analysis are presented in Tables S4–S15.

Table 2: Private and social profits of chili farming in research locations, dry season of 2019 and rainy season of 2019/2020

Description		Financial (private) profits (IDR)	Economic (social) profits (IDR)
A	***Wet-lowland agroecosystem***		
1	**Kediri**		
	a. Dry season	41,568,450	98,425,650
	b. Rainy season	25,971,446	83,599,372
2	**Tabalong**		
	a. Dry season	39,732,170	19,369,121
	b. Rainy season	30,428,835	13,848,843
3	**Bantaeng**		
	a. Dry season	34,829,813	47,059,067
	b. Rainy season	23,823,563	45,657,282
B	***Dry-upland agroecosystem***		
1	**Bandung**		
	a. Dry season	71,460,774	90,860,423
	b. Rainy season	58,278,225	79,009,226
2	**Banjarnegara**		
	a. Dry season	37,502,785	35,422,076
	b. Rainy season	22,348,570	30,695,257
3	**Tanah Karo**		
	a. Dry season	70,347,662	91,861,438
	b. Rainy season	48,541,037	107,393,438

3.2 Competitive and comparative advantages

Competitiveness can be viewed from two standpoints: financial (private) competitiveness and social or economic competitiveness. Financial (private) competitiveness refers to competitive advantage, whereas social or economic competitiveness refers to comparative advantage. According to the analysis results, chili farming in chili production centers in Indonesia is competitive on both wet-lowland and dry-upland agroecosystems. PCR coefficient values <1 and DRCR values <1 show that chili farming in Indonesia's production centers has both competitive and comparative advantages.

At private prices, the PCR value of <1 means less than a unit of domestic resource cost is needed to produce one unit of value-added output. The DRCR value of <1 indicates that less than one unit of foreign exchange is required for domestic resources to save one unit of foreign exchange. In most of the research locations, the competitive advantage is higher than the comparative advantage. In all research locations, the chili competitiveness in the dry season is lower than in the rainy season. The values of PCR <1 and DRCR <1 imply that chili farming in Indonesia has both competitive and comparative advantages.

The PCR and DRCR values show that chili farming in the Bandung district has the highest competitiveness (competitive and comparative advantages) in all the seasons, indicated by the lowest PCR and DRCR values. The PCR value is 0.513 in the dry season and 0.548 in the rainy season. Meanwhile, the DRCR value is 0.448 in the dry season and 0.467 in the rainy season. In contrast, chili

farming in the Banjarnegara district shows the lowest competitive advantage, with PCR values at 0.689 in the dry season and 0.783 in the rainy season. The highest DRCR coefficient values, meaning the lowest comparative advantage, are found in Tabalong district. It is 0.814 in the dry season and 0.853 in the rainy season. The resulting DRCR values follow those reported by Salam and Tufail's [58]. However, it is not valid for the PCR values. Another study on chili competitiveness in Banten [59] shows very low PCR and DRCR values, 0.208 and 0.113, respectively, indicating high competitiveness of chili farming in the corresponding location. Table 3 presents the PCR and DRCR values for chili farming by agroecosystem and location in Indonesia.

If compared with other commodities, the competitiveness of chili is lower than that of snake fruit and rice. Pronojiwo snake fruit farming has a PCR value of 0.13 and a DRCR value of 0.20 [22], whereas rice shows a PCR value of 0.12 and a DRCR value of 0.27 [23]. The most competitive commodity is nutmeg, with a DRCR value of 0.05 and a PCR value of 0.05 [60]. In comparison, China's wheat has DRCR values >1 in all provinces [61], showing that the system has no comparative advantage. This indicates that utilizing local resources is more costly than using global resources.

Table 3: PCR and DRCR coefficient values of chili farming in research locations, dry season of 2019 and rainy season of 2019/2020

Description		PCR	DRCR
A	***Wet-lowland agroecosystem***		
1	**Kediri**		
	a. Dry season	0.677	0.462
	b. Rainy season	0.744	0.469
2	**Tabalong**		
	a. Dry season	0.688	0.814
	b. Rainy season	0.740	0.858
3	**Bantaeng**		
	a. Dry season	0.685	0.606
	b. Rainy season	0.751	0.601
B	***Dry-upland agroecosystem***		
1	**Bandung**		
	a. Dry season	0.513	0.448
	b. Rainy season	0.548	0.467
2	**Banjarnegara**		
	a. Dry season	0.689	0.697
	b. Rainy season	0.783	0.720
3	**Tanah Karo**		
	a. Dry season	0.597	0.527
	b. Rainy season	0.670	0.488

All abbreviations are given in Table 1.

3.3 Impact analysis of divergency and government policy

In PAM analysis, output transfers (OT), input transfers (IT), factor transfers (FT), and net transfers (NT) are the absolute measures of the impact of divergence or government policy on chili farming in production centers. Meanwhile, the nominal protection coefficient on output (NPCO), nominal protection coefficient on inputs (NPCI), effective protection coefficient (EPC), profitability coefficient (PC), and subsidy ratio to producers (SRP) reflect the relative measures.

3.3.1 Impact of government policy on outputs

The OT and NPCO values show the policy impact on chili output in production center areas in Indonesia. Trade policies, such as export taxes, value-added taxes (VAT), import tariffs, subsidies, and supportive policies, are examples of government policies in the output sector. The gap between revenues computed using private prices and those derived using social prices is known as OT. The NPCO is a measure of OT, defined as the ratio of revenues computed using private prices to revenues computed using social prices.

Table 4 presents the OT and NPCO values of chili farming in production center areas in Indonesia. The

Table 4: OT and NPCO values of chili farming in research locations, dry season of 2019 and rainy season of 2019/2020

Description		OT (IDR)	NPCO
A	***Wet-lowland agroecosystem***		
1	**Kediri**		
	a. Dry season	−48,643,496	0.771
	b. Rainy season	−49,291,560	0.739
2	**Tabalong**		
	a. Dry season	32,442,260	1.248
	b. Rainy season	24,628,060	1.192
3	**Bantaeng**		
	a. Dry season	−6,361,080	0.953
	b. Rainy season	−15,989,232	0.876
B	***Dry-upland agroecosystem***		
1	**Bandung**		
	a. Dry season	−6,842,745	0.964
	b. Rainy season	−8,370,149	0.951
2	**Banjarnegara**		
	a. Dry season	9,141,342	1.065
	b. Rainy season	−841,960	0.994
3	**Tanah Karo**		
	a. Dry season	−17,965,520	0.925
	b. Rainy season	−62,482,520	0.756

All abbreviations are given in Table 1.

results of the analysis show variations between locations and between agroecosystems. Negative OT values and NPCO >1 are found in Kediri, Bantaeng, Bandung, and Tanah Karo districts in the dry and rainy seasons and Banjarnegara district in the rainy season. Meanwhile, positive OT values and NPCO >1 are found in Tabalong district in both seasons and Banjarnegara district in the dry season. Hence, in most locations, chili farming indicates negative OT values with the NPCO coefficient >1, indicating that chili farmers receive actual prices lower than they should in a no distortion market. Distortion happens to the market because of government policy intervention or market distortion. This finding reveals that farmers face disincentives in chili farming on the output side. This finding aligns with Tinaprilla's study (Tinaprilla 2008) in Bandung and Bandung Barat districts. The results of an empirical study and a literature review show that the market structure of chili in production centers in Indonesia is oligopsony. It is characterized by numerous sellers and fewer buyers [62]. Such a condition causes little competition among chili traders [63].

Chili farming in the wet-lowland of Tabalong district shows the highest positive NPCO values, i.e., 1.248 in the dry season and 1.192 in the rainy season. In contrast, chili farming in the wet-lowland of Kediri district indicates the lowest NPCO values, i.e., 0.771 in the dry season and 0.739 in the rainy season. These findings mean that chili farmers in Tabalong district have an incentive to produce from the output side, whereas chili farmers in other locations experienced a disincentive. Chili farmers in Kediri district experience the most significant disincentive in chili farming. In general, the analysis results show that most chili farmers received a lower selling price than they should. This phenomenon is in contrast with the case of China's wheat, where the farmers receive higher prices than global prices because of the existing policy [61].

3.3.2 Impacts of government policy on inputs

The IT and NPCI values illustrate the government policy impact on tradable inputs for chili farming in Indonesia. Meanwhile, the FT value indicates the impact of government policy or divergences on domestic factors.

The government policy imposed on tradable inputs and domestic factors can be in trade policies (export taxes, import tariffs), input subsidies, and VAT. At the same time, market distortions can cause other forms of divergence, either because of market failure or market structures that are not perfect. IT show the difference between the costs of tradable inputs at the private and social prices. The NPCI is an input transfer indicator, the ratio between tradable input costs calculated based on private prices and social prices. Table 5 presents the values of IT, NPCI, and FT indicators in chili farming at the research sites.

In all research locations, chili farming provides positive IT values. Similarly, the NPCI coefficient value is >1 in most of the research locations. Farmers have disincentives on the tradable input side because they must pay higher input prices than in a perfectly competitive market, as indicated by positive IT values and NPCI values >1. The NPCI values in chili farming are the highest in Bandung district (1.411 in the dry season, 1.458 in the rainy season). In contrast, the lowest NPCI values are experienced in Tanah Karo district (1.033 in the dry season, 1.001 in the rainy season). This means that, in general, there are tradable input policies that are harmful to chili farmers because they are forced to pay tradable input prices higher than in a perfect market. The source of the distortion is suspected to be tariffs and VAT. At the same time, subsidies for production facilities have been partially eliminated, and empirically, the price of non-subsidized chemical fertilizers is much higher than that of subsidized fertilizers.

The analysis results show that the FT values of chili farming in most research locations are positive, except in

Table 5: IT, NPCI, and FT values of chili farming in research locations, dry season of 2019 and rainy season of 2019/2020

	Description	IT (IDR)	NPCI	FT (IDR)
A	***Wet-lowland agroecosystem***			
1	**Kediri**			
	a. Dry season	5,887,174	1.203	2,326,530
	b. Rainy season	1,523,034	1.033	2,025,222
2	**Tabalong**			
	a. Dry season	8,985,725	1.335	3,093,486
	b. Rainy season	24,628,060	1.192	5,137,467
3	**Bantaeng**			
	a. Dry season	2,497,200	1.162	3,370,974
	b. Rainy season	2,538,750	1.179	3,305,737
B	***Dry-upland agroecosystem***			
1	**Bandung**			
	a. Dry season	10,932,177	1.411	1,624,727
	b. Rainy season	10,901,755	1.458	1,459,097
2	**Banjarnegara**			
	a. Dry season	5,453,079	1.225	1,607,554
	b. Rainy season	5,917,250	1.239	1,587,477
3	**Tanah Karo**			
	a. Dry season	1,523,034	1.033	2,025,222
	b. Rainy season	27,984	1.001	−3,658,103

All abbreviations are given in Table 1.

Tanah Karo district in the rainy season. Tabalong district has the highest FT values, at IDR 3,093,486/ha/season in the dry season and IDR 5,137,467/ha/season in the rainy season. Meanwhile, the lowest FT value is discovered in Tanah Karo district during the rainy season, at −IDR 3,658,103/ha/season. These findings indicate the presence of government policy intervention or market distortion in domestic factors that are harmful to chili farmers because they must pay higher domestic factor prices than they should. A capital interest is the primary source of price differences in domestic factor costs. Farmers pay more than social prices, but the differences are small.

3.3.3 Impact of government policy on input–output

The values of NT, EPC, PC, and SRP indicate the impact of divergence and government policies on overall inputs and outputs of chili farming in Indonesia. Table 6 shows the findings of the impact analysis of divergence and government policies on the input and output of chili farming in Indonesia's chili production centers.

The analysis results show that the EPC values of chili farming in both agroecosystems in all research locations are positive and >0. The highest positive EPC values are demonstrated in Tabalong district in the dry and rainy seasons, 1.226 and 1.200, respectively. In contrast, the lowest positive EPC values are in Kediri district, at 0.702 in the dry season and 0.645 in the rainy season. These findings show that government policies or market distortions occurring in chili farming, in general, are disincentives to chili farmers. Hence, most chili farmers do not obtain effective protection from existing policies or market distortions, except in Tabalong district in both dry and rainy seasons and Banjarnegara district in the dry season. This finding is in line with the study result of Haryanto and Masyhuri [20], who assess the competitiveness of corn farming in marginal areas. Their study shows that the government's protection of agricultural outputs is regarded as weak, contrary to tradable and non-tradable agricultural inputs. In addition, the government's strategy on agricultural outputs and inputs does not properly safeguard corn cultivation.

Chili farmers face disincentives as a result of government policies or market distortions in the industry as a whole. This means that, with the exception of Tabalong district in both the dry and rainy seasons and Banjarnegara district in the dry season, most chili farmers do not receive effective protection from existing policies or market distortions than perfectly competitive market mechanisms.

The magnitude of the NT values in chili farming in production center areas in Indonesia is mainly negative, except in Banjarnegara district in the dry season and Tabalong district in the dry and rainy seasons. In the rainy season, chili farming in Tanah Karo district has the largest negative NT value at −IDR 58,852.401/ha/season. In comparison, chili farming in Tabalong district in the dry season has the largest positive NT value at IDR 20,363,049/ha/season. Government policy or market distortions imposed on tradable inputs and domestic factors and overall outputs are mostly disincentives to chili farmers in general. This means that farmers, as producers, are more likely to make large transfers to consumer groups.

The results of the PC analysis are positive. However, in most research locations, PC value is <1, except in Banjarnegara district in the dry season and Tabalong district in both the dry and rainy seasons. The PC value <1 indicates that the chili farmers' profits are smaller than if the market is in perfect competition. The highest PC value occurs in chili farming in Banjarnegara district in the dry season at 1059. In contrast, the lowest PC value occurs in chili farming in the rainy season in Kediri district at 0.311. Farmers face disincentives as a result of government policies or market distortions in the chili industry as a whole. This makes most chili farmers get lower profits than in a perfect market.

Table 6: NT, PC, EPC, and SRP values of chili farming in research locations, dry season of 2019 and rainy season of 2019/2020

	Description	EPC	NT (IDR)	PC	SRP
A	***Wet-lowland agroecosystem***				
1	**Kediri**				
	a. Dry season	0.702	−56,857,200	0.422	−0.268
	b. Rainy season	0.645	−57,627,926	0.311	−0.306
2	**Tabalong**				
	a. Dry season	1.226	20,363,049	2.051	0.156
	b. Rainy season	1.200	16,579,992	2.197	0.129
3	**Bantaeng**				
	a. Dry season	0.926	−12,229,254	0.740	−0.091
	b. Rainy season	0.838	−21,833,719	0.522	−0.170
B	***Dry-upland agroecosystem***				
1	**Bandung**				
	a. Dry season	0.892	−19,399,649	0.786	−0.102
	b. Rainy season	0.870	−20,731,001	0.738	−0.121
2	**Banjarnegara**				
	a. Dry season	1.032	2,080,709	1.059	0.015
	b. Rainy season	0.938	−8,346,687	0.728	−0.062
3	**Tanah Karo**				
	a. Dry season	0.900	−21,513,776	0.766	−0.089
	b. Rainy season	0.702	−58,852,401	0.452	−0.230

All abbreviations are given in Table 1.

In most research locations, the SRP values in chili farming in both agroecosystems obtained a negative SRP coefficient value, except in Tabalong district in both seasons and Banjarnegara district in the dry season. The magnitude of the SRP value of chili farming with the largest negative sign occurs in Kediri Regency in the dry (−0.268) and rainy (−0.306) seasons. On the other hand, chili farming in Tabalong district has the highest positive SRP value at 0.156 in the dry season and 0.129 in the rainy season. Thus, government policies or market distortions in chili farming, in general, have a negative impact on chili farmers, causing them to experience negative subsidies.

4 Conclusions

Chili farming in Indonesia is profitable both privately and socially. On average, farmers' private profits are lower than their social profits, indicating that chili farmers in Indonesia face disincentives in producing chili. Incentive policies are needed to encourage farmers to keep planting chili by ensuring a more competitive market mechanism for inputs and outputs, especially the output market. The output price policy can be carried out through guaranteed output prices by optimizing the function of the Farmers Market as a captive market for chili farmers.

Chili farming in Indonesia's production centers has competitive and comparative advantages. Therefore, increasing domestic chili production is more beneficial for Indonesia than importing chili from other countries because of the efficient use of domestic resources. Policies to increase chili production in production center areas can be carried out by facilitating farmers to access production facilities (seed, fertilizers, pesticides), capital assistance, and marketing.

Government policies and market distortions in the input and output sectors, on the whole, are less beneficial for chili farmers. Therefore, the relevant government policy is to increase an efficient production and logistics system with the support of agricultural infrastructure, distribution, and marketing facilities, increase access to market information, eliminate inefficiencies, and encourage competitive market mechanisms.

Overall, this study suggests that the government should develop chili farming in production centers with an integrated agribusiness area approach. In addition, policies to encourage the availability of hybrid chili seeds, rational fertilizer subsidy programs, increase access to credit and agricultural insurance, and encourage the operation of market mechanisms to run competitively are also needed.

Supplementary materials: The online version of this article contains supplementary materials available at doi: 10.1515/opag-2022-0083.

Acknowledgments: The authors thank the District Offices of Agriculture and other government authorities as well as the farmer groups at the research sites.

Funding information: The field survey and primary data collection related to this research were supported by the Directorate General of Horticulture, Ministry of Agriculture.

Author contributions: S.S. – conceptualization, data curation, investigation, methodology, validation, and writing – original draft; E.A. – conceptualization, methodology, writing – original draft, and writing review – review and editing; A.A. – conceptualization, methodology, writing – original draft, and writing review – review and editing; E.G. – data curation and formal analysis; A.D.P. – formal analysis, validation, and writing – original draft; S.G.S. – formal analysis, validation, and writing – original draft; H.P.S. – formal analysis, validation, and writing review – review and editing; H.J.P. – methodology and writing review – review and editing; K.S.I. – validation and writing review – review and editing; A.D.P. – validation and writing – original draft; N.Q.H. – data curation, investigation, and writing review – review and editing.

Conflict of interest: The authors state no conflict of interest.

Data availability statement: The datasets generated during and/or analyzed during the current study are available from the corresponding author on reasonable request.

References

[1] Riswati F, Warsito I. Challenge for ASEAN-China free trade agreement on Indonesia employment. Econ J Emerg Mark. 2010;2(1):89–100.

[2] Bouët A. The expected benefits of trade liberalization for world income and development: opening the "black box" of global trade modeling. Food policy review 8. Washington, DC: International Food Policy Research Institute; 2008. doi: 10.2499/0896295109FPREV8.

[3] Gultom D. Regional comprehensive economic partnership (RCEP): how Indonesia can maximize the opportunities, an

insider's perspective. Policy Brief No. 6. Jakarta: Center for Indonesian Policy Studies; 2020.
[4] Friyatno S, Saptana S. Kinerja agribisnis komoditas pertanian: kemampuan penciptaan output, nilai tambah dan keterkaitan antar sektor (Analisis komparasi I-O tahun 2005 dan 2010). J Manaj Agribisnis. 2017;14(3):250–63. doi: 10.17358/jma.14.3.250.
[5] Ingot SR, Laksani DD. Indonesia global value chain participation in regional comprehensive economic partnership (RCEP). Adv Econ Manag Res. 2019;98:159–63. doi: 10.2991/icot-19.2019.34.
[6] Kaplinsy R. Globalization and unequalization: what can be learned from value change analysis. J Dev Stud. 2010;37(2):117–46.
[7] Banga R Measuring value in global value chains. Background Paper No. RVC-8. Geneva: Unit of Economic Cooperation and Integration amongst Developing Countries, United Nations Conference on Trade and Development; 2013.
[8] Gereffi G, Fernandez-Stark K. Global value chain analysis: a primer. Durham: Center on Globalization, Governances and Competitiveness, Duke University; 2016.
[9] United Nations Industrial Development Organization. Global value chains and development. New York: UNIDO Publishing; 2015.
[10] World Trade Organization. Global value chain development report 2017: measuring and analyzing the impact of GVCs on economic development. Washington DC: World Bank Publishing; 2017.
[11] World Bank. World development report 2020: trading for development in the age of global value chains. Washington DC: World Bank Publishing; 2020.
[12] Saptana S. Tinjauan konseptual mikro-makro daya saing dan strategi pembangunan pertanian. 2010;28(1):1–18. doi: 10.21082/fae.v28n1.2010.1-18.
[13] Gaytán D, Benita F. On the competitiveness of Mexico's dry chili production. Econ Agric. 2014;61(2):307–17. doi: 10.22004/ag.econ.175284.
[14] Wiranthi PE, Mubarok F. Competitiveness and the factors affecting export of the Indonesia canned pineapple in the world and the destination countries. In: Muhaemin M, Hidayat Y, Lengkey HAW, editors. Proceedings of the 2nd International Conference on Sustainable Agriculture and Food Security: A Comprehensive Approach. Sumedang: KnE Life Sciences; 2017. p. 339–52. doi: 10.18502/kls.v2i6.105.
[15] Notta O, Vlachvei A. Competitiveness in food and beverage manufacturing industries. In: Tsounis N, Vlachvei A, Gaetano M, Monovasilis T, editors. Proceedings: International Conference on Applied Economics. Kozani: TEI of Western Macedonia Press; 2011. p. 437–44.
[16] Firmansyah F, Widodo W, Karsinah K, Oktavilia S. Export performance and competitiveness of Indonesian food commodities. J Ekon Kebijak. 2017;10(2):289–310. doi: http://dx.doi.org/10.15294/jejak.v10i2.11294.
[17] Ministry of Agriculture. Outlook cabai. Ministry of agriculture. Jakarta: Center For Data and Information System; 2020.
[18] Monke EA, Pearson SR. The policy analysis matrix for agricultural development. Ithaca: Cornell University Press; 1998.
[19] Pearson PS, Bahri, Gostch C. Is rice probability in Indonesia still profitable? FPSA Working Paper, February 2005. California: Stanford University; 2005.
[20] Haryanto LI, Masyhuri M, Irham I. The policy analysis matrix in measuring competitiveness of maize farming system in marginal areas. Agro Ekon. 2018;2:244–60. doi: 10.22146/ae.35964.
[21] Winarno ST, Harisudin M. Competitiveness analysis of robusta coffee in East Java, Indonesia. Acad Strateg Manag J. 2018;17(6):1–9.
[22] Soetriono D, Maharani DA, Zahrosa DB. The competitiveness of Pronojiwo snake fruit. E3S Web Conf. 2020;142:05007. doi: 10.1051/-e3sconf/202014205007.
[23] Poernomo A. Analysis of the protection of input subsidies policy (fertilizer and seed) and production output in rice plant agriculture in Indonesia. Eko-Regional. 2017;12(1):49–55.
[24] Falatehan AF, Syaukat Y, Hastuti, Hardjanto A, Puspitosari DA. Determining comparative and competitive advantages of Indonesian tobacco through policy analysis matrix (PAM). Agric Sci. 2018;1(2):75–83.
[25] Ugochukwu AI, Ezedinma CI. Intensification of rice production systems in southeastern Nigeria: a policy analysis matrix approach. Int J Agric Manag Dev. 2011;1(2):89–100. doi: 10.22004/ag.econ.143498.
[26] Kanaka S, Chinnadurai M. The policy analysis matrix of rice cultivation in India. Eur J Basic Appl Sci. 2015;2(1):33–45.
[27] Souza ARL, Révillion JPP, Waquil PD, Belarmino LC, Lanfranco BA. Economic and accounting evaluation of rice milled production chain in Rio Grande do Sul (Brazil) and Uruguay with application of the policy analysis matrix. Ciência Ruaral. 2017;47(4):1–7. doi: 10.1590/0103-8478cr20151085.
[28] Lazzarini SG. Strategizing by the government: can industrial policy create firm level competitive advantage. Strateg Manag J. 2015;36:97–112. doi: 10.1002/smj.2204.
[29] Farla K. Industrial policy for growth. J Ind Compét Trade. 2015;15:257–82. doi: 10.1007/s10842-014-0183.
[30] Paltasing KR, Balekere BWTO. and Indian chilli export: issue of growth, instability and competitiveness. Indian J Agric Mark. 2014;28(2):35–50.
[31] Sativa M, Harianto, Suryana A. Impact of red chilli reference price policy in Indonesia. Int J Agric Syst. 2017;5(2):120–39. doi: 10.20956/ijas.v5i2.1201.
[32] Anggorowati SD, Sugiyarto, Darwanto DH. Strategies for agribusiness development of chili farming in Boyolali regency. J Agribus Manag Dev. 2021;2(1):68–75.
[33] Tinaprilla N. Analisis daya saing dan kebijakan pemerintah pada usahatani cabai merah (Kasus Kecamatan Ciwidey Kabupaten Bandung dan Kecamatan Lembang Kabupaten Bandung Barat). J Agribisnis Ekon Pertan. 2008;2(2):39–64.
[34] Rum M. Analisis usahatani dan kebijakan pemerintah terkait cabai merah besar di Kabupaten Malang dengan menggunakan policy analysis matrix (PAM). Embrio. 2010;7(2):138–43.
[35] Kusuma RL, Firdaus M. Daya saing dan faktor yang mempengaruhi volume ekspor sayuran Indonesia terhadap negara tujuan utama. J Manaj Agribisnis. 2015;12(3):226–36. doi: 10.17358/jma.12.3.226.
[36] Hamdani H, Salawati U, Nuryadin MR. Agribusiness competitiveness and potential growth of cabai hiyung in Tapin District, South Kalimantan. In: Soendjoto MA, Dharmono, Riefani MK, editors. Prosiding Seminar Nasional Lahan Basah. Jilid 1: Potensi, Peluang, dan Tantangan Pengelolaan. Banjarmasin: Lambung Mangkurat University Press; 2016. p. 156–63.

[37] Astining CA, Bangun RH. Karakteristik petani dan kelayakan usahatani cabai besar (*Capsiccum annuum* L) dan cabai rawit (*Capsiccum frutescens* L) di Sumatera Utara. Agricore. 2020;5(1):49–58.
[38] Indrasti R, Rawung JBM, Sudolar NR, Andri KB, Sahram, Tan SS. Farmer institutional and economic feasibility study on red chilli pepper farming in Kepung district, Kediri regency, East Java province. IOP Conf Ser Earth Env Sci. 2021;807:022018. doi: 10.1088/1755-1315/807/2/022018.
[39] Analia D. Struktur rantai pasok (*supply chain*), kelembagaan dan klaster industri komoditas cabai: sebuah tinjauan literatur. Agripita. 2017;1(1):21–30.
[40] Arhim M, Putri DA, Nurlaela R, Anisa A, Rukka MR, Risamasu PIM, et al. Supply chain management performance toward competitiveness of chili as main agriculture commodity. IOP Conf Ser Earth Env Sci. 2019;343:012108. doi: 10.1088/1755-1315/343/1/012108.
[41] Firdaus M, Suherman, Wahyudi F, Sauqi A, Widaninggar N. The factors driving and inhibiting the large chili agribusiness in Jember Regency. J Adv Res Dyn Control Syst. 2020;12(1):193–200. doi: 10.5373/JARDCS/V12I1/20201029.
[42] Tsurayya S, Kartika L. Kelembagaan dan strategi peningkatan daya saing komoditas cabai Kabupaten Garut. J Manaj Agribisnis. 2015;12(1):1–13. doi: 10.17358/jma.12.1.1.
[43] Mariyono J. Marketing channels of chili: players, levels, and segmentations. J Econ Business Acc Ventur. 2019;22(2):167–76. doi: 10.14414/jebav.v22i2.1688.
[44] Saidah Z, Harianto H, Hartoyo S, Asmarantaka RW. Transaction cost analysis on revenues and profits of red chili farming. J Manaj Agribisnis. 2019;16(1):66–6. doi: 10.17358/jma.16.1.66.
[45] Quddus MA, Mustafa U. Comparative advantage of major crops production in Punjab: an application of policy analysis matrix. Lahore J Econ. 2011;16(1):63–94.
[46] Gunawan E, Perwita AD, Sukmaya SG, Darwis V, Ariningsih E. The competitiveness analysis of shallot in Indonesia: A policy analysis matrix. PLoS ONE. 2021;16(9):e0256832. doi: 10.1371/journal.pone.0256832.
[47] Gupta SD. Comparative advantage and competitive advantage: an economics perspective and a synthesis. Athens J Bus Econ. 2015;1(1):9–22. doi: 10.30958/ajbe.1-1-.
[48] Karyani T, Susanto A, Djuwendah E, Hapsari H. Red chili agribusiness and the risks faced by the farmers. IOP Conf Ser Earth Env Sci. 2020;466:012002. doi: 10.1088/1755-1315/466/1/012002.
[49] Andayani SA, Sumekar Y, Sukmawati R, Ismail AY, Nugraha DR, Umyati S. Increasing liquidity of SSDM-based red chili farmers through agricultural insurance. Jordan J Biol Sci. 2021;14(2):353–8.
[50] Mariyono J, Daroini PB, Dewi HA, Latifah E. Comparative study of socioeconomic chili farming between East Java and Bali. J Sos Ekon Pertan. 2018;11(2):36–45.
[51] Hidayah KA. Analisis finansial usahatani cabai merah skala petani di Kota Samarinda (Studi kasus di Kelurahan Lempake Samarinda). J Agrifor. 2014;13(1):1–10.
[52] Ridiyanto T, Soetoro, Hardiyanto T. Analisis usahatani cabai merah (Capsicum annum L.)varietas Hot Beauty (Studi kasus di Desa Sukamaju Kecamatan Cihaurbeuti Kabupaten Ciamis). Agroinfo Galuh. 2017;4(2):132–9.
[53] Agnes A, Antara M. Analisis pendapatan dan kelayakan usahatani cabai rawit di Desa Sunju Kecamatan Marawola Kabupaten Sigi. e-J Agrotekbis. 2017;5(1):86–91.
[54] Nofita I, Sutiarso E, Hadi S. Analisis keuntungan usahatani cabai merah besar di Desa Andongsari Kecamatan Ambulu Kabupaten Jember. Agritrop. 2015;13(2):166–71.
[55] Eliyatiningsih MayasariF Factors that influence farmers' decision to keep his red chili farming in Wuluhan district, Jember regency. In: Wahono A, Pantaya D, Riskiawan HY, Shahbaz H, Indarto I, Kim EJ, editors. Proceeding of the 1st International Conference of Food and Agriculture. Jember: Politeknik Negeri Jember; 2018. p. 55–61.
[56] Puspitasari A. Analisis biaya dan pendapatan usahatani cabai rawit di Kecamatan Cigalontang Kabupaten Tasikmalaya. Mimb Agribisnis. 2020;6(2):1130–42.
[57] Geo L, Ariani WOR, Saediman. Determinants and profitability of small-scale red chili production in Konawe district of Southeast Sulawesi. IOSR J Agric Vet Sci. 2020;13(3):51–5.
[58] Salam A, Tufail S. Competitiveness and comparative advantage of important food and industrial crops in Punjab: application of policy analysis matrix. J Int Agric Trade Dev. 2016;10(1):81–94.
[59] Siagian V. Impact increasing input and output price toward competitiveness of chili in Banten province. In: Maharijaya A, editor. Proceedings of the International Seminar on Tropical Horticulture Horticulture for the Quality of Life. Bogor: Pusat Kajian Hortikultura Tropika LPPM – IPB; 2018. p. 249–60.
[60] Assagaf M, Musyafak A, Wahab A, Hermawati Cahyaningrum H, Amiruddin A. The competitiveness analysis of nutmeg farming as spices in Ternate, North Moluccas. IOP Conf Ser Earth Env Sci. 2021;807:042077. doi: 10.1088/1755-1315/807/4/042077.
[61] Saad A, Zhang R, Xia Y. The policy analysis matrix (PAM): comparative advantage of China's wheat crop production 2017. J Agric Sci. 2019;11(17):150–7.
[62] InterCAFE, JICA. Market study on food sector in Indonesia. Final report. Bogor: International Center for Applied Finance and Economics, Lembaga Penelitian dan Pengabdian Masyarakat; 2018.
[63] Wardhono A, Indrawati Y, Qori'ah CG, Nasir MA. Institutional arrangement for food price stabilization and market distribution system: study of chili commodity in Banyuwangi regency. E3S Web Conf. 2020;142:05006. doi: 10.1051/e3sconf/202014205006.
