# Supplementary material for: Competitiveness analysis of fresh tomatoes in Indonesia: Turning comparative advantage into competitive advantage
Source: PLoS One. 2023 Nov 30;18(11):e0294980. doi: 10.1371/journal.pone.0294980 (PMC10688876; doi:10.1371/journal.pone.0294980)
Supplement: Attachment — Submitted filename: Response to Reviewers.docx [file pone.0294980.s004.docx]

**Competitiveness Analysis of Fresh Tomato in Indonesia: Turning Comparative Advantage into Competitive Advantage**

**Reviewers' comments:**

Reviewer's Responses to Questions

Comments to the Author

1) Is the manuscript technically sound, and do the data support the conclusions?

The manuscript must describe a technically sound piece of scientific research with data that supports the conclusions. Experiments must have been conducted rigorously, with appropriate controls, replication, and sample sizes. The conclusions must be drawn appropriately based on the data presented.

Reviewer #1: Yes

Reviewer #2: Partly

**Response:**

The total sample was 15 farmer households for each district in Java Island and 10 farmer households for each district in the Outer Java region. The sample selection of farmer households represents large land farmers, medium land farmers and small land farmers. Where farmers in each class use relatively homogeneous technology, data and information are obtained from agricultural extension workers and district agricultural officers.

Besides that, Focus Group Discussions (FGD) were also conducted at each research location which consisted of farmer group leaders and administrators, farmer/advanced farmer leaders, field agricultural extension officers, district agriculture service officers, village collectors, traders between regions, wholesalers. / wholesaler and exporter.

The conclusions have been corrected and supported by data analysis results.

2) Has the statistical analysis been performed appropriately and rigorously?

Reviewer #1: No

**Response:**

This study uses a mathematical approach (Policy Analysis Matrix), not a statistical approach so it does not require a certain number of respondents (N), but emphasizes the strength of the respondents' representation. Respondents were selected purposively to represent the biophysical and socioeconomic variations in each study location.

Reviewer #2: Yes

3) Have the authors made all data underlying the findings in their manuscript fully available?

The PLOS Data policy requires authors to make all data underlying the findings described in their manuscript fully available without restriction, with rare exception (please refer to the Data Availability Statement in the manuscript PDF file). The data should be provided as part of the manuscript or its supporting information, or deposited to a public repository. For example, in addition to summary statistics, the data points behind means, medians and variance measures should be available. If there are restrictions on publicly sharing data—e.g. participant privacy or use of data from a third party—those must be specified.

Reviewer #1: Yes

Reviewer #2: Yes

4) Is the manuscript presented in an intelligible fashion and written in standard English?

PLOS ONE does not copyedit accepted manuscripts, so the language in submitted articles must be clear, correct, and unambiguous. Any typographical or grammatical errors should be corrected at revision, so please note any specific errors here.

Reviewer #1: No

**Response:**

The language structure has been improved to make it clearer and correct. Typical and minor errors have been corrected and refined.

Reviewer #2: Yes

5) Review Comments to the Author

Please use the space provided to explain your answers to the questions above. You may also include additional comments for the author, including concerns about dual publication, research ethics, or publication ethics. (Please upload your review as an attachment if it exceeds 20,000 characters)

Reviewer #1: I concent to the sampling of the study location and respondents. (1) What justified you chose 6 district for the study location?. please clarity the location related the representative of the tomato center and farmers. Is it represent or not?. (2). Then you used the FGD to data collection and only 10 farmers. I think its not enough and need more sample. (3) Please change your recommendation follow your objective and more implemented. (4) Please check the similarity index

Dear Reviewer #1. We thank you for your valuable comments. Please find our answers to your inquiries as the following:

1) here are six study locations selected in this research, consisting of 3 regencies on Java Island namely: (1) Bandung Regency representing the West Java production center area; (2) Banjarnegara Regency representing the Central Java Province production center area, (3) Kediri Regency representing the East Java production center area and 3 other regencies outside Java Island namely; (4) Tanah Karo Regency representing production centers in North Sumatra, (5) Tabalong Regency representing production centers in the developing South Kalimantan Province, and (6) Pinrang Regency representing production centers in South Sulawesi. BPS data shows that reflected tomato production in Java contributed 464,865.4 tons (41.71%), Sumatra Island contributed 392,964.2 tons (35.26%), Kalimantan Island contributed 27,789 tons (2.49%), and South Sulawesi contributed 156,608.5 tons (14.07%) of Indonesia's total production of 1,114,399.5 tonnes (100%). Furthermore, the locations were chosen based on whether they had exported tomatoes or had the potential to export tomatoes. As a result, the author believes that the research locations chosen are representative of the performance of tomato production in Indonesia.
2) This study did not only use the FGD method, but also a limited survey: (i) for farmer respondents, there were 15 respondent regencies in Java and 10 respondent regencies for areas outside Java Island; (ii) In this research conducted a limited survey was also carried out through interviews with traders at various levels, both collecting traders as many as 2, traders between regions 2 and wholesalers 2 and retailers 2 people; and (iii) in addition, farmer group leaders and farmer group administrator, field agricultural extension workers, field extension coordinators, Agriculture Service officers in each sample district, traders, packing house entrepreneurs, and exporters participated in FGDs, with approximately 10 FGD participants per sample district.
3) In addition to statistical analysis, this study uses mathematical analysis which does not require a certain number of samples as in statistical analysis. However, the selected sample has been attempted to be representative of each location. Thus the research sample totaling 10-15 farmer respondents and supplemented by FGDs attended by groups of farmers and farmers, extension workers, Agricultural Service officers, collectors, wholesalers, and exporters (10 respondents) have represented all farmers and business actors in the research area.
4) The similarity index was calculated, and the result is 10%.

Reviewer #2: The manuscript focuses on the actual topic - financial and economic profitability of tomato farming. Authors applied competitive and comparative advantages and assessed the impact of government policies. Finally, they suggests some strategic policies to create comparative advantage into its competitive advantage in the market. The manuscript is quite inconsistent and very extensive and meandering. On the other side, I miss detail discussion.

Authors should considers following recommendations:

I appreciate deep analyses of literature reviewed however, I have some small comments to improve the manuscript. Literature Review is extensive. Some parts should be consider as methodology, (e.g. Line 212-220) (have changed to line 429-436) and some part are not directly interlinking with following research (e.g. Line 271-283) (have been erased). Often it discusses a topic that is not directly related to the research results. (e.g. Line 106-114 How is interlinking a characterisation of tomato nutritional composition with the topic of research?) (have been added a linking sentences in line 112-116)

On the other side some part of Methodology could be considered as Literature Review – (Line 287-310) (Have been changed to Literatur Review Part (line 167-190).

Chapter “Results” also consists some theoretical descriptions, which should be move as methodology part. (e.g. Line 549-569) (have been changed in ke line 397-418)

In results describes claims that are speculative, without substantiated research. (e.g. Line 516: In general, the research findings suggest that the value of the competitive advantage coefficient is higher than the benefit of the comparative advantage….. Could you in detail explain sentences like that?) (has been corrected and added with the alignment of the results of previous studies in Spain and the Netherlands, data is also shown in table 4, at line 565-572)

On the other side, some parts (like Discussion) should be described in more detail. The authors could easily implement the discussion as a separate part of the manuscript, to compare their results with similar research (should be mentioned in Literature Review) and highlight scientific novelty of the study.

It is necessary to supplement your conclusions with the knowledge of other researches. Moreover, some of your conclusion and suggestion could be considered as speculation, because there are not directly interlinking with results. (e.g. Line 702-709) (have been erased in the main article)

Additionally, the conclusion must better highlighted novelty and the scientific significance of the results.

**Dear Reviewer #2. We thank you for your detail examination on our paper. Please find our answers to your inquiries as the following:**

**Response:**

1) Harmonized the whole section of the Literature Review, Methodology, and Results

**Response:**

We have harmonized the whole section of the Literature Review, Methodology, and Results as you suggested. The new body of text has improved the quality of this paper. By restructuring sentences, removing some irrelevant literature, and adding the most recent relevant literature, improvements have been made in a more systematic and focused manner.

Note: Besides the FGD there is a limited survey that needs to be explained in the methodology

2) Literature Review is extensive

**Response:**

The literature has been simplified by removing literature that is not directly related to the topic and adding literature that is related.

3) Some parts should be consider as methodology

**Response:**

The literature on theory and concepts has been moved to the methodology chapter and literature that is empirical review has been included in the Literature Review chapter.

4) Some part are not directly interlinking with following research (e.g. Line 271-283)

**Response:**

The authors agreed that non-related literature should be excluded and partially replaced with related literature and the latest.

5) Often it discusses a topic that is not directly related to the research results.

**Response:**

The authors agreed that non-related literature should be excluded.

6) How is interlinking a characterisation of tomato nutritional composition with the topic of research?

**Response:**

The connection between tomato composition characterization and competitiveness can be explained as follows: market demand conditions describe consumer demand for goods and services produced by producers. Current conditions These, global consumer demand necessitates more comprehensive and detailed product attributes such as safety, nutritional, value, attributes, ecolabel, product traceability dan humanistic.

Borrowing the term introduced (Porter, 1990) sophisticated and demanding buyers, namely consumers who are intelligent and critical will provide valuable information to producers regarding the quality of products and services needed by consumers.

7) On the other side some part of Methodology could be considered as Literature Review – (Line 287-310). (have been Changed to line 167-190)

**Response:**

Empirical research literature has been moved from the methodology section to the literature review section, and vice versa, the literature which is a theoretical conceptual review has been transferred to the methodology section.

8) Chapter “Results” also consists some theoretical descriptions, which should be move as methodology part. (e.g. Line 549-569) (have been Changed to metodologi part in line 397-418)

**Response:**

The authors agreed to move the competitiveness literature review, which is a theoretical and conceptual review, to the methodology section.

9) In results describes claims that are speculative, without substantiated research. (e.g. Line 516: In general, the research findings suggest that the value of the competitive advantage coefficient is higher than the benefit of the comparative advantage….. Could you in detail explain sentences like that?)

**Response:**

These findings are not speculative because the findings of this study show consistent results across regions and seasons. Furthermore, to support these findings, the results of other researchers' studies have been reviewed in the literature. To show that this study is not speculative, it is necessary to show the figures in the table that the general level of PCR competitiveness in several locations is greater than the DRCR value.

10) On the other side, some parts (like Discussion) should be described in more detail. The authors could easily implement the discussion as a separate part of the manuscript, to compare their results with similar research (should be mentioned in Literature Review) and highlight scientific novelty of the study.

**Response:**

It has been completed with a relevant literature review, comparative analysis was carried out with the results of studies by other researchers, and has been synthesized. Article status and scientific updates have been completed.

11) It is necessary to supplement your conclusions with the knowledge of other researches. Moreover, some of your conclusion and suggestion could be considered as speculation, because there are not directly interlinking with results. (e.g. Line 702-709).

**Response:**

The writing team may consider suggestions to focus on answering research objectives and referencing the findings of other researchers' studies, but some sections are directly related to research methodology, such as the allocation of the distribution of cost components into tradable inputs and domestic factors, as well as the calculation of social prices, and private budged and social budget are the stages and calculations of PAM.

12) The conclusion must better highlighted novelty and the scientific significance of the results.

**Response:**

The novelty in this research includes: (i) this research was conducted across regions and across seasons so that it can more comprehensively represent the biophysical and socioeconomic diversity of farmers and tomato business actors in the production site compared to other studies that are case studies or partial; (ii) provide novelty that consistently that the value of the DRCR (comparative advantage) coefficient is lower than the PCR (competitive advantage) coefficient value which means that the competitive advantage from the social (economic) perspective is higher than the competitive advantage from the private (financial) perspective; (iii) provide novelty to the tomato commodity system in Indonesia that the effects of divergence caused by government policies and overall input-output market errors are more favorable to consumers than producers; and (iv) provide a policy strategy perspective to realize comparative advantage into competitive advantage through appropriate and equitable government policies between producers and consumers.

**Note:** It is necessary to show how many areas were used in this study and what percentage of production compared to the total national tomato production. In addition, it is also necessary to add the number of other areas that are not used in research along with the percentage of production to the total national tomato production. It is necessary to add information about Bandung's share of West Java, the share of Karo Land against North Sumatra etc.

Scientific significance of research results: (i) Provide an understanding of the use of PAM analysis tools and the stages that must be carried out (allocation of tradable input components and domestic factors, calculation of social prices, farm budget private perspective and social perspective), and finally calculate PAM indicators; (ii) Provide understanding in narrating the results of profitability analysis privately (financially) and socially (economic), competitive advantages and comparative advantages as well as the impact of divergence caused by government policies and market failure; and (iii) Formulate appropriate and equitable government policy strategies not only for consumers but also for producers.

Recommendations The results of this study can be used as input to synergize and harmonize with existing policies to be used as an improvement in tomato commodity development policies nationally.

**Note:** For example, as a recommendation to reduce PPH for horticultural agricultural products, improve oligopsonistic market structure, etc.
