# Supplementary material for: Competitiveness analysis of fresh tomatoes in Indonesia: Turning comparative advantage into competitive advantage
Source: PLoS One. 2023 Nov 30;18(11):e0294980. doi: 10.1371/journal.pone.0294980 (PMC10688876; doi:10.1371/journal.pone.0294980)
Supplement: Attachment — Submitted filename: Response to Reviewers.docx [file pone.0294980.s005.docx]

**Competitiveness Analysis of Fresh Tomato in Indonesia: Turning Comparative Advantage into Competitive Advantage**

**Reviewers' comments:**

Reviewer's Responses to Questions

Comments to the Author

1. If the authors have adequately addressed your comments raised in a previous round of review and you feel that this manuscript is now acceptable for publication, you may indicate that here to bypass the “Comments to the Author” section, enter your conflict of interest statement in the “Confidential to Editor” section, and submit your "Accept" recommendation.

Reviewer #3: (No Response)

2. Is the manuscript technically sound, and do the data support the conclusions?

The manuscript must describe a technically sound piece of scientific research with data that supports the conclusions. Experiments must have been conducted rigorously, with appropriate controls, replication, and sample sizes. The conclusions must be drawn appropriately based on the data presented.

Reviewer #3: Partly

**Response:**

We have added improvements to the sampling method and conclusions section according to the reviewer's instructions. in the sampling method section we make improvements by providing a more detailed explanation, this can be seen in lines 342-354 and 365-376. then we have also made improvements to the conclusion section, this can be seen in lines 772-776.

3. Has the statistical analysis been performed appropriately and rigorously?

Reviewer #3: Yes

4. Have the authors made all data underlying the findings in their manuscript fully available?

The PLOS Data policy requires authors to make all data underlying the findings described in their manuscript fully available without restriction, with rare exception (please refer to the Data Availability Statement in the manuscript PDF file). The data should be provided as part of the manuscript or its supporting information, or deposited to a public repository. For example, in addition to summary statistics, the data points behind means, medians and variance measures should be available. If there are restrictions on publicly sharing data—e.g. participant privacy or use of data from a third party—those must be specified.

Reviewer #3: Yes

5. Is the manuscript presented in an intelligible fashion and written in standard English?

PLOS ONE does not copyedit accepted manuscripts, so the language in submitted articles must be clear, correct, and unambiguous. Any typographical or grammatical errors should be corrected at revision, so please note any specific errors here.

Reviewer #3: Yes

6. Review Comments to the Author

Please use the space provided to explain your answers to the questions above. You may also include additional comments for the author, including concerns about dual publication, research ethics, or publication ethics. (Please upload your review as an attachment if it exceeds 20,000 characters)

Reviewer #3: The authors have adequately responded to questions and comments of previous reviewers. Consequently, believe the manuscript amidst the corrections is favorable for publication.

Again, the manuscript has a strong literature and methodology which served as the basis of its foundation. Technically one can consider the manuscript as sound and the data duly corresponds to the conclusion made. However, the justification for the sample size is not clear.

Also, the authors may have to re-read the manuscript to correct a few omissions and incomplete sentences. For example, in paragraphs 304 and 305, "On the other hand, countries with relatively abundant and scarce labor will tend to export labor-intensive products and import capital intensive products" There is an omission in-between "abundant and and" which I believe it is "capital". Moving to paragraphs 486 and 487, "In general, economic benefits (social) were higher than financial benefits (private), profits during rainy season were lower those during dry season". I believe here also that, there is a letter omission "than" in-between "lower and those".

Dear Reviewer #3. We thank you for your detailed examination on our paper. Please find our answers to your inquiries as the following:

**Response:**

1) The justification for the sample size is not clear.

**Response:**

We have added improvements to the sampling method and conclusions section according to the reviewer's instructions. In the sampling method section we make improvements by providing a more detailed explanation, this can be seen in lines 342-354 and 365-376. then we have also made improvements to the conclusion section, this can be seen in lines 772-776.

2) Re-read the manuscript to correct a few omissions and incomplete sentences.

**Response**:

We have corrected omissions and incomplete grammar according to the direction of the reviewer. Our fixes are on lines 25, 32-33, 74, 182-184, 272-274, and 524-525.

7. PLOS authors have the option to publish the peer review history of their article (what does this mean?). If published, this will include your full peer review and any attached files.

If you choose “no”, your identity will remain anonymous but your review may still be made public.

Do you want your identity to be public for this peer review? For information about this choice, including consent withdrawal, please see our Privacy Policy.

Reviewer #3: No
