# Supplementary material for: Competitiveness analysis of fresh tomatoes in Indonesia: Turning comparative advantage into competitive advantage
Source: PLoS One. 2023 Nov 30;18(11):e0294980. doi: 10.1371/journal.pone.0294980 (PMC10688876; doi:10.1371/journal.pone.0294980)
Supplement: Attachment — Submitted filename: Review PONE-D-22-29206R2.docx [file pone.0294980.s006.docx]

1. Please avoid unnecessary explanations such as “Tomato (Lycopersicon esculentum mill) is a widely known vegetable in Indonesia” as this is not important in the context of the manuscript.
2. The manuscript needs to be edited by a professional English editor.
3. Need more rationales in the introduction section, why it is important to assess the comparative and competitive advantages.
4. Please revise the abstract to make it more interesting align with the findings and recommendations and explain more about the samples in the abstract.
5. Explore more about the findings, not only about the number but also the critical analysis. If a tomato has comparative and competitive advantages, so what? What is the rationale behind it, and what does it imply? what is the next?
6. The recommendations are not sound, please make them clear and more applicable.
7. The conclusion is different from a summary, so please refine it.
8. We found that the authors previously wrote similar ideas and topics, but in different commodities, so please explain the differences and the novelty, not only in different commodity.
